# VDAC1 is a target for pharmacologically induced insulin hypersecretion in β cells

**DOI:** 10.1016/j.celrep.2025.115834

**Published:** 2025-06-11

**Authors:** Gitanjali Roy, Andrea Ordóñez, Derk D. Binns, Karina Rodrigues-dos-Santos, Michael B. Kwakye, George C. King, Rachel L. Kuntz, Noyonika Mukherjee, Andrew T. Templin, Zhiyong Tan, Timothy I. Richardson, Emma H. Doud, Amber L. Mosley, Kathryn L. Schueler, Christopher H. Emfinger, Alan D. Attie, Mark P. Keller, Travis S. Johnson, Michael A. Kalwat

**Affiliations:** 1 Indiana Biosciences Research Institute, Indianapolis, IN, USA; 2 Department of Cell Biology, University of Texas Southwestern Medical Center, Dallas, TX, USA; 3 Center for Diabetes and Metabolic Diseases, Indiana University School of Medicine, Indianapolis, IN, USA; 4 Department of Biostatistics, Indiana University School of Medicine, Indianapolis, IN, USA; 5 Department of Pharmacology and Toxicology, Indiana University School of Medicine, Indianapolis, IN, USA; 6 Department of Biochemistry and Molecular Biology, Indiana University School of Medicine, Indianapolis, IN, USA; 7 Department of Medicine, Indiana University School of Medicine, Indianapolis, IN, USA; 8 Roudebush VA Medical Center, Indianapolis, IN, USA; 9 Center for Proteome Analysis, Indiana University School of Medicine, Indianapolis, IN, USA; 10 Center for Computational Biology and Bioinformatics, Indiana University School of Medicine, Indianapolis, IN, USA; 11 Department of Biochemistry, University of Wisconsin-Madison, Madison, WI, USA; 12 Lead contact

## Abstract

β cells are dysfunctional in type 2 diabetes (T2D) and congenital hyperinsulinism (HI), but the mechanisms linking hypersecretion to β cell failure are poorly understood. Here, we use proteomics and functional assays in human and mouse β cell lines to identify VDAC1 as a target for the small molecule hypersecretion inducer SW016789. By enhancing membrane depolarization, SW016789 acutely increases Ca^2+^ influx, eventually driving β cell dysfunction. Time-course transcriptomics analysis reveals a distinct hypersecretory response signature compared to classical endoplasmic reticulum (ER) stress, highlighting ER-associated degradation (ERAD) as a key adaptive pathway. While SW016789 reduces ERAD substrate OS-9 levels, broader ERAD component changes are limited in cell lines. However, immunostaining of the T2D human pancreas shows altered distributions of the ratios of the core ERAD components SEL1L, HRD1, and DERL3 in β cells. This work provides a detailed mechanistic characterization of a hypersecretion-specific stress response, revealing potential therapeutic targets, including VDAC1 and ERAD, for modulating β cell function and survival in disease.

## In brief

Roy et al. identify VDAC1 as a target for pharmacologically induced insulin hypersecretion in β cells. Hypersecretory stress causes a distinct transcriptomic signature that implicates ER-associated degradation (ERAD) as an adaptive response. The relative abundance of ERAD components is altered in pancreatic islets of type 2 diabetic humans.

## Graphical Abstract

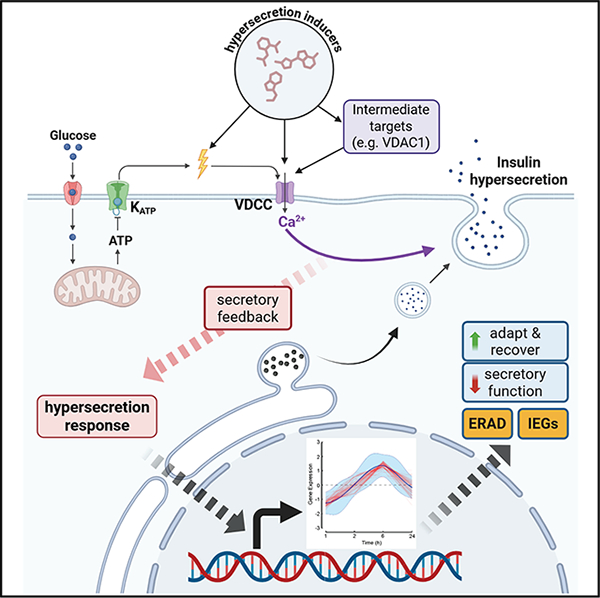

## INTRODUCTION

Pancreatic β cells must secrete insulin to maintain glucose homeostasis. Failures in this process lead to type 2 diabetes (T2D).^1^ In T2D, impaired production of and response to insulin leads to chronic hyperglycemia and cardiovascular comorbidities. β cells normally sense increased circulating glucose concentrations through passive uptake and subsequent glycolytic and mitochondrial metabolism, followed by the secretion of insuin, which travels to peripheral tissues to stimulate glucose clearance. ^2,3^ These metabolic events in the β cell involve oscillations in [ATP] and [ADP], with ATP stimulating closure and ADP stimulating opening of ATP-sensitive potassium channels (K_ATP_). ^4^ K_ATP_ closure leads to membrane depolarization, voltage-dependent Ca^2+^ channel (VDCC) opening, and Ca^2+^ influx, which triggers insulin exocytosis. ^5–11^ Concurrently, metabolic amplification augments exocytosis without further Ca^2+^ influx. ^12–14^ While insulin secretion is necessary for glucose homeostasis, this process can go awry when secretory rates are elevated inappropriately (insulin hypersecretion) in rare diseases like congenital hyperinsulinism. Insulin hypersecretion may also contribute to prediabetes, T2D, and some cases of monogenic diabetes via multiple mechanisms. ^15–18^ We define the hypersecretory response as β cell dysfunction occurring due to an abnormally high secretory rate caused by pharmacological, environmental, or genetic factors. Insulin hypersecretion can occur before the development of insulin resistance. ^19,20^ However, the mechanisms behind insulin hypersecretion and the associated responses remain incompletely understood. It is also unknown to what extent insulin hypersecretion may instigate β cell failure and which steps might be therapeutically targetable. In some cases, diabetes treatments that promote secretion can exacerbate β cell dysfunction. ^21–23^ However, hypersecretion responses can be reversible, suggesting the possibility of therapeutic interventions. ^21,24^ To prevent β cell dysfunction in T2D and other genetic diseases, therapies are needed to alleviate hypersecretion while maintaining glucose homeostasis. Hyperglycemia can also drive β cell failure independent of insulin hypersecretion, ^25^ further supporting the need for multiple treatment strategies.

Insulin hypersecretion may present as repeated occurrences for short durations or chronically elevated secretory rates and is known to be induced by multiple small molecules. ^21,24,26–28^ In response to these treatments, we and others have found that β cells adapt by halting secretory function. ^21,24,26^ The mechanisms of pharmacologically induced insulin hypersecretion and its downstream impacts on β cell function are not fully understood. We previously identified a small molecule, SW016789, which acutely causes insulin hypersecretion (1–2 h) and results in loss of β cell function after longer exposure (4–24 h). ^24^ We determined that SW016789 enhanced nutrient-stimulated Ca^2+^ influx to elicit hypersecretion, subsequently causing a transient endoplasmic reticulum (ER) stress-like response and loss of secretion, but without cell death. We also found that blocking Ca^2+^ influx protected against the loss of function caused by chronic SW016789 exposure. We concluded that the acute and chronic effects of hypersecretion are inextricably linked and not actually two opposite activities. The proximal mechanism of action for SW016789 and the nature of the β cell hypersecretory response were unclear.

In the current study, we report the discovery of a target protein for SW016789 activity, the voltage-dependent anion channel VDAC1. Further, we find that hypersecretion can occur in response to structurally diverse compounds and that the key steps likely lie downstream of enhanced Ca^2+^ influx. To better understand the processes involved, we used time-course transcriptomics and clustering analyses to elucidate a unique hypersecretory response signature. This signature is enriched for multiple pathways, including core components of ER-associated degradation (ERAD), coat protein complex II (COP-II) transport, serine and one-carbon metabolism, and certain ER chaperones. We found that ERAD may play a part in the β cell adaptive response to hypersecretory stress to avoid cell death and that the ratio between ERAD components may be altered in islets in T2D.

## RESULTS

### SW016789 induces hypersecretory stress in human β cells and interacts with VDAC1

**Figure 1.**
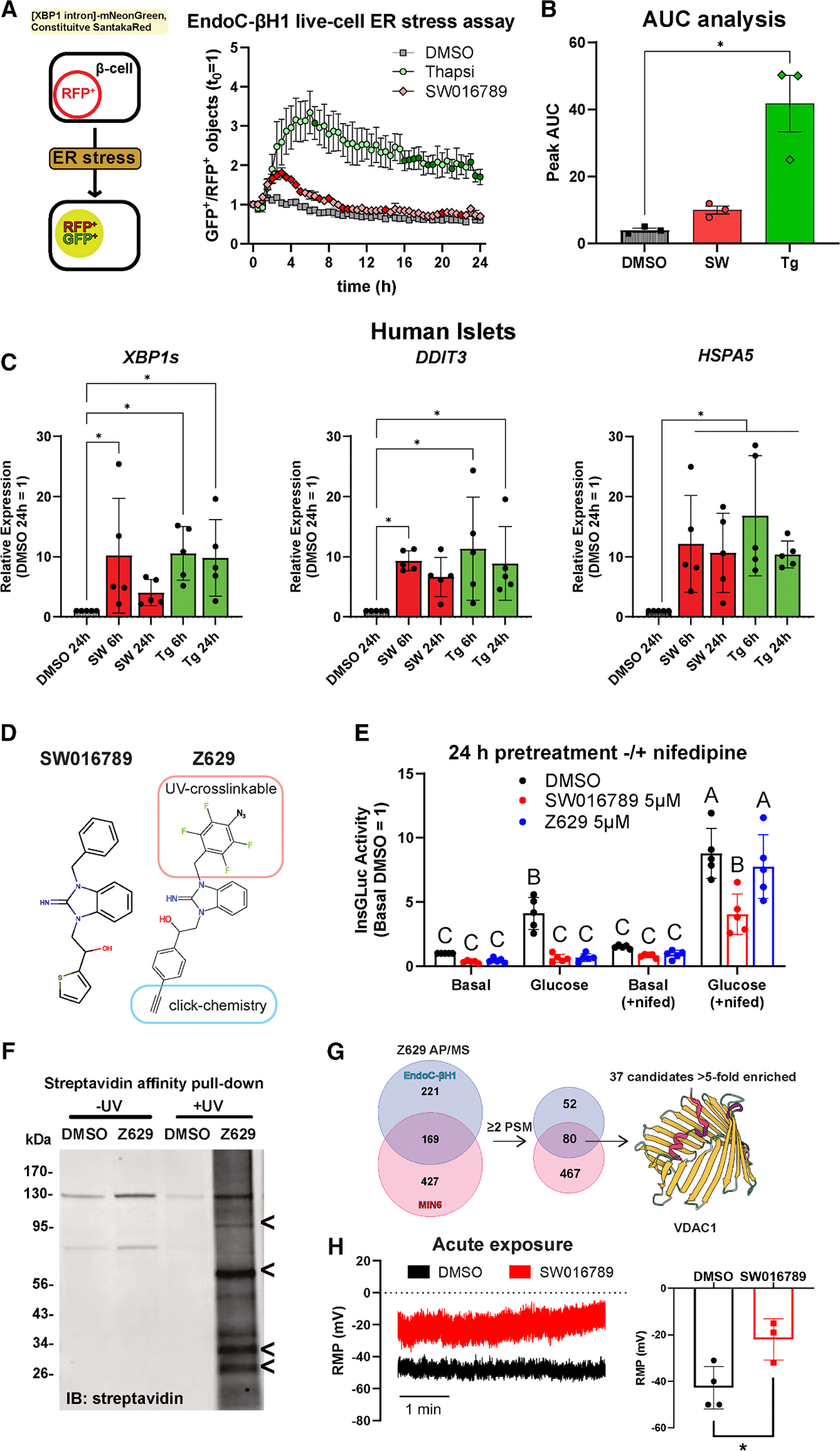
VDAC1 is implicated in hypersecretion induced by SW016789 in human and mouse β cells (A) SW016789-treated (10 μM) and Tg-treated (3 μM) EndoC-β1 cells expressing a fluorescent XBP1 splicing-based ER stress sensor. Data are the mean ± SE of *n* = 3. Differences are assessed by two-way repeated measures ANOVA with Dunnett’s multiple comparisons test. Data points with *p* < 0.05 vs. DMSO are indicated by a darker fill color (SW016789 [SW], dark red; Tg, dark green). (B) Area under the curve (AUC) analysis for (A). Data are the mean ± SE of *n* = 3. *p < 0.05 vs. DMSO by one-way ANOVA with Dunnett’s multiple comparisons test. (C) Gene expression in human islets treated for 6 or 24 h with SW (10 μM) or thapsigargin (Tg; 1 μM) or DMSO (0.1%) for 24 h. Data are the mean ± SD of *n* = 5. *p < 0.05 vs. DMSO by one-way ANOVA with Dunnett’s multiple comparisons test. (D) Chemical structure of SW and the photoaffinity probe Z629. (E) InsGLuc secretion assay in β cells treated for 24 h with DMSO, SW (5 μM), or Z629 (5 μM) in the presence or absence of nifedipine (10 μM). Data are the mean ± SD of *n* = 5. Differences (*p* < 0.05 by two-way ANOVA with Tukey’s multiple comparison test) are indicated by labeling with different letters. (F) Streptavidin blot indicating biotin-conjugated proteins in MIN6 cells. (G) Candidate targets of SW overlapping between MIN6 and EndoC-βH1 cells. Candidates among the 37 hits in Table S2 include *DYNC1H1*, *PCSK1*, *VDAC1*, *VDAC2*, *VDAC3*, *CKAP4*, *SLC3A2*, and *SEC22B*. (H) Resting membrane potential measurements in MIN6 cells treated with DMSO (0.1%) or SW (5 μM). Data are the mean ± SD of *n* = 3–4. *p < 0.05 by unpaired t test. See also Figure S1.

We previously published the phenotypic effects of SW016789-induced hypersecretory stress in β cells.^24^ However, the exact pathways through which hypersecretion acts are not well understood. Hypersecretion was hypothesized to activate the unfolded protein response of the ER (UPR^ER^). One of the pathways that can activate the UPR^ER^ is the IRE1α-mediated splicing of Xbp1 mRNA. To test whether SW016789-induced hypersecretion could activate the UPR^ER^ in human EndoC-βH1 cells, we used an XBP1 splicing-based reporter. SW016789 caused a transient and relatively low-amplitude ER stress response compared to the larger and sustained response elicited by the known ER stressor thapsigargin (Tg), which acts by depleting ER Ca^2+^ (Figures 1A and 1B). The transient nature of hypersecretory stress from SW016789 compared to sustained ER stress from thapsigargin was consistent in isolated human islets treated for either 6 or 24 h, as shown by spliced *XBP1* and *DDIT3* (CHOP) expression (Figure 1C). However, *HSPA5* (BiP) expression was sustained with both treatments. Our previous findings indicated that SW016789-induced hypersecretory stress depended on Ca^2+^ influx, but the exact protein target(s) were unknown. To address this, we leveraged structure-activity relationship data using a small set of chemical analogs of SW016789.^24^ Based on the activity of those analogs, we determined that the central hydroxyl group could not be modified and that adding side chains may be tolerated on the benzyl and thiophene rings. We designed a photoaffinity probe, Z629, to perform direct target identification experiments (Figures 1D and S1A). To verify that Z629 retained activity, we treated InsGLuc-MIN6 cells for 24 h in the presence of DMSO, SW016789, or Z629. We observed that both SW016789 and Z629 suppressed glucose-stimulated secretion (Figure 1E). We have shown previously that the dihydropyridine VDCC blocker nifedipine protects against the inhibitory effects of SW016789.^24^ Using this paradigm, we observed that nifedipine also protected β cells against Z629 (Figure 1E). Notably, nifedipine was more effective at protecting against Z629 compared to SW016789. This may be due to the structural modifications of the Z629 probe causing slightly reduced potency as a hypersecretion inducer compared to the original compound. However, these data indicated that Z629 has activity similar to the parental SW016789 compound. The Z629 probe incorporates an alkyne for click chemistry and a tetrafluorophenyl azide for UV crosslinking. Having established SW016789 activity in mouse and human β cell lines, we performed target identification studies in both MIN6 and EndoC-βH1 β cells to increase the chances of identifying relevant targets. We treated both lines with Z629 or DMSO and exposed the cells to UV-B light. The lysates were subjected to click chemistry and affinity purification followed by immunoblotting to confirm biotin labeling (Figure 1F). Bands present in the non-UV-treated samples are endogenously biotinylated proteins (e.g., pyruvate carboxylase, ~130 kDa). The experiment was repeated, and samples were subjected to on-bead digestion for proteomics analysis. We identified 80 potential Z629-interacting proteins overlapping between MIN6 and EndoC-βH1 β cells, with ≥2 spectral counts in the Z629-treated samples (Figure 1G, Table S1). Applying a >5-fold enrichment cutoff narrowed this list to 37 candidates. VDAC1, VDAC2, and VDAC3 were detected in Z629 pull-down from both MIN6 and EndoC-βH1 cells, consistent with their expression in both cell lines and in human islets (Figures S1B and S1C). Across multiple endocrine cell lines, VDAC1 gene expression was the highest in MIN6 β cells (Figure S1D). VDAC1 also stood out among the candidates because it has been implicated in the β cell in T2D and has the potential to alter membrane potential, which may explain the effects of SW016789.^29^ Supporting this, we observed increased resting membrane potential in MIN6 cells exposed to SW016789 (Figure 1H).

### VDAC1 is a direct target of SW016789 and is necessary for full SW016789 activity in β cells

**Figure 2.**
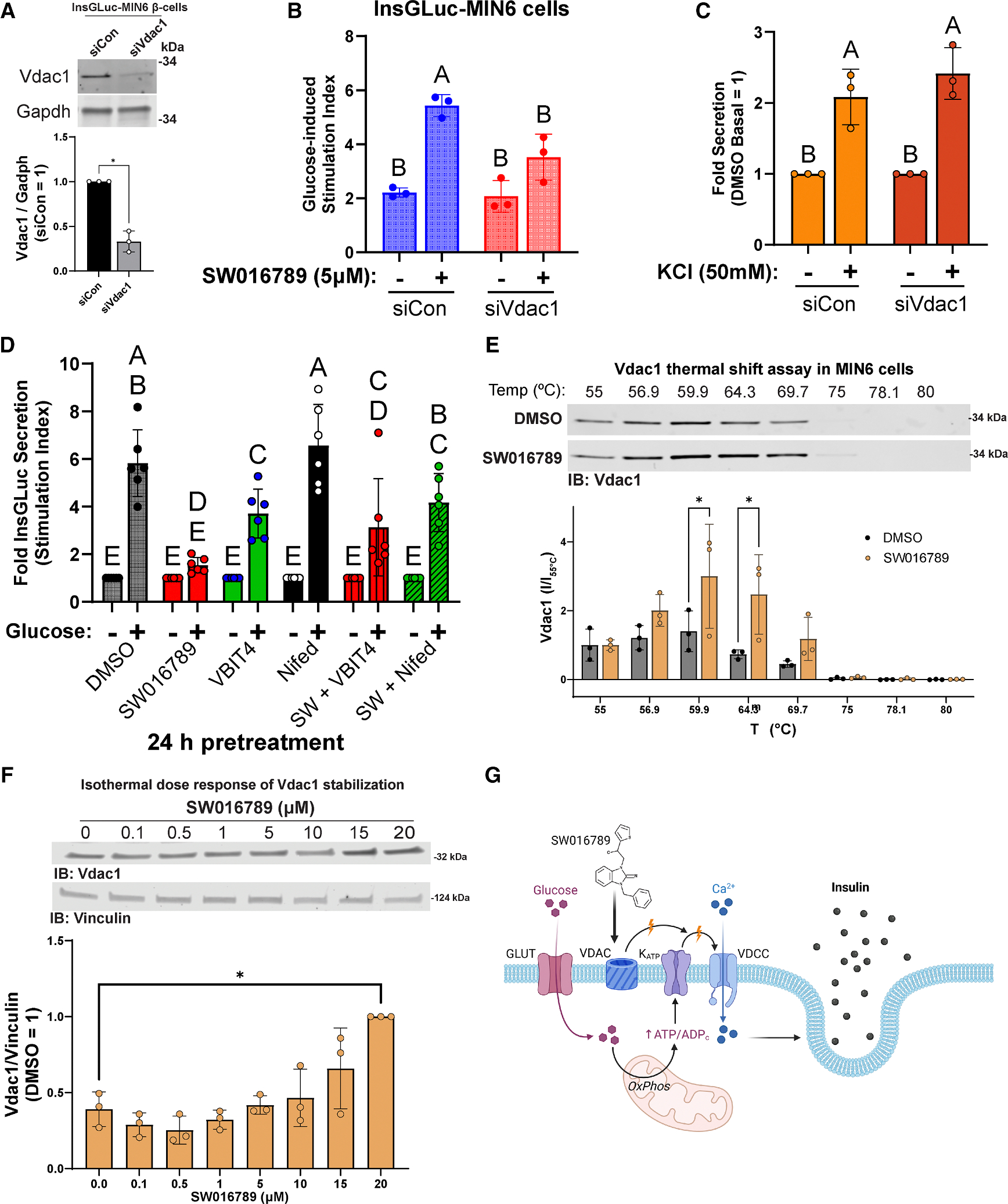
VDAC1 is stabilized by and required for SW activity (A) Depletion of *Vdac1* in InsGLuc-MIN6 β cells. Data are the mean ± SD of *n* = 3. *p < 0.05 by unpaired t test. (B) Glucose (20 mM) stimulated insulin secretion in *Vdac1*-depleted InsGLuc-MIN6 cells co-treated with 5 μM SW016789 (SW). Data are the mean ± SD of *n* = 3. Differences (*p* < 0.05 by two-way ANOVA with Tukey’s multiple comparison test) are indicated by different letters. (C) KCl-induced secretion in *Vdac1*-depleted InsGLuc-MIN6 cells. Data are the mean ± SD of *n* = 3. Differences (*p* < 0.05 by two-way ANOVA with Tukey’s multiple comparison test) are indicated by labeling with different letters. (D) 24 h treatment with SW (5 μM) in the presence or absence of the VDAC1 inhibitor VBIT4 (10 μM) or nifedipine (10 μM). Data are the mean ± SD of *n* = 6. Differences (*p* < 0.05 by two-way ANOVA with Tukey’s multiple comparison test) are indicated by labeling with different letters. (E) VDAC1 immunoblot from a cellular thermal shift assay (CETSA) in MIN6 β cells treated with SW. Data are the mean ± SD of *n* = 3. *p < 0.05 by two-way ANOVA with Sidak’s multiple comparisons test. (F) Isothermal dose-response CETSA for SW in MIN6 β cells. Data are the mean ± SD of *n* = 3. *p < 0.05 by one-way ANOVA with Dunnett’s multiple comparisons test. (G) Working model of SW actions through VDAC1. See also Figure S2.

To validate VDAC1 as a target, we applied three orthogonal methods. First, we depleted *Vdac1* in InsGLuc-MIN6 β cells using small interfering RNA (Figure 2A). *Vdac1*-depleted cells had a significantly reduced response to acute SW016789-enhanced glucose-stimulated secretion (Figures 2B and S2A). However, KCl-stimulated secretion was intact in *Vdac1*-depleted cells (Figure 2C), suggesting that membrane depolarization and secretion induced solely by Ca^2+^ influx were not impaired. Second, we co-treated cells for 24 h with SW016789 alone or in combination with the VDAC1 inhibitor VBIT4 or nifedipine. Treatment with SW016789 alone inhibited subsequent glucose-stimulated secretion, as expected (Figure 2D). ^24^ However, co-treatment of SW016789 with either VBIT4 or nifedipine protected against this inhibition (Figure 2D). Accordingly, VBIT4 suppressed the enhancement of glucose-stimulated Ca^2+^ influx induced by SW016789 (Figure S2B). Third, we applied the thermal shift assay (CETSA), which uses a temperature gradient to assess alterations in the melting temperature of the protein of interest upon binding of a ligand. ^30^ We exposed MIN6 β cells to a temperature gradient of 37 °C–75 °C to determine endogenous VDAC1 aggregation in the absence of ligand. Immunoblotting shows the stability of VDAC1 at temperatures up to 71 °C (Figure S2C). Next, we shifted to a higher temperature gradient (55 °C–80 °C) in the presence of DMSO or SW016789 (10 μM). SW016789 caused a significant shift in VDAC1 stability at 59.9°C–64.3 °C compared to DMSO (Figures 2E and S2D). Isothermal concentration dependence of SW016789 (0–20 μM) on VDAC1 stability was then determined at 64.3°C in MIN6 cells. VDAC1 stability appeared to increase in response to increasing concentrations of SW016789 and was increased significantly at 20 μM SW016789 (Figure 2F). Based on these data, we conclude that SW016789 can act through VDAC1 to cause hypersecretion, possibly via increasing membrane ion permeability and raising membrane potential, thereby potentiating glucose-stimulated Ca^2+^ influx (Figure 2G).

### Small molecule-mediated hypersecretion-induced loss of β cell function *in vitro* requires chronic Ca^2+^ influx

**Figure 3.**
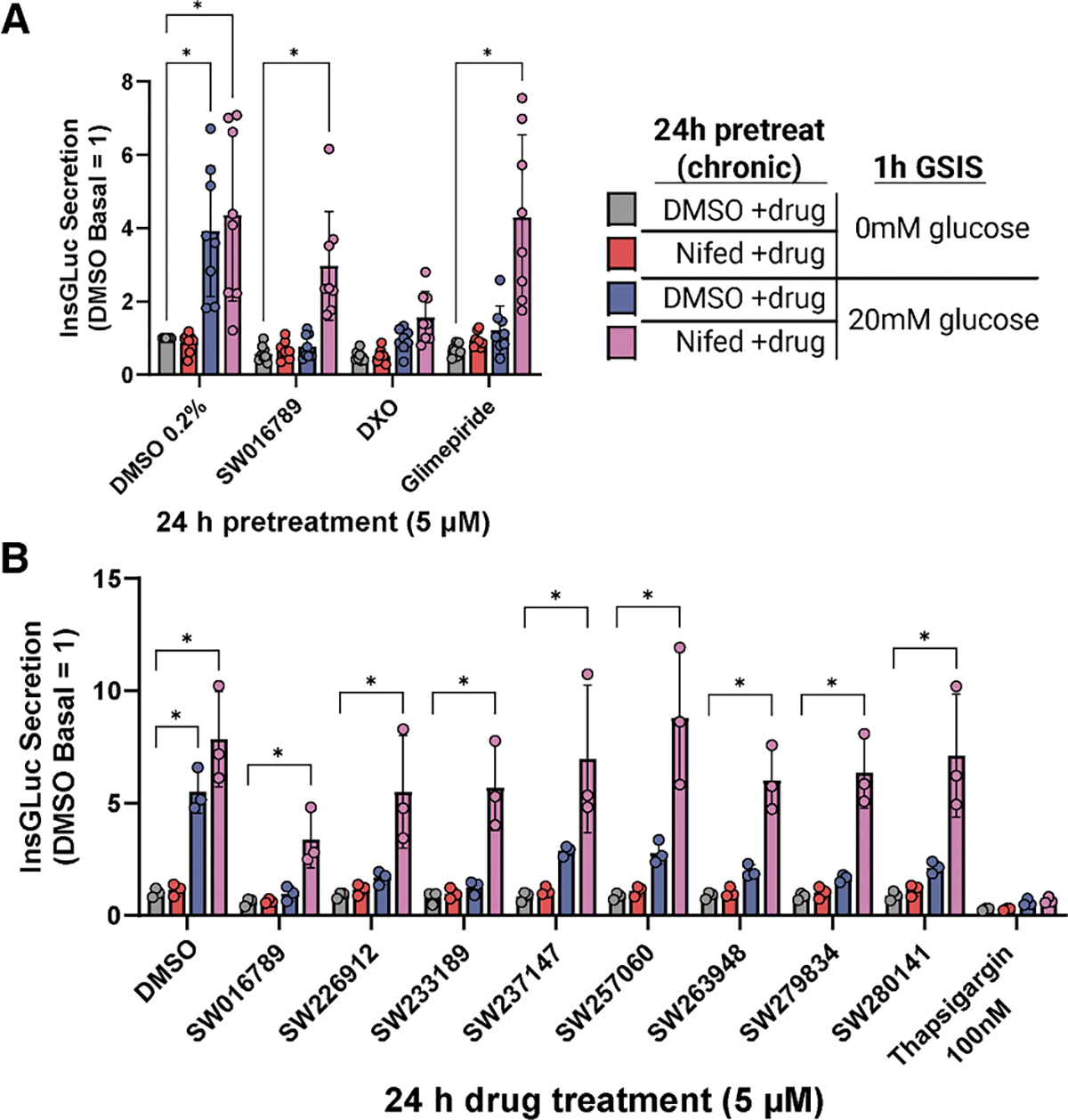
Ca^2+^ influx mediates the loss of β cell function in response to small-molecule hypersecretion inducers (A) Glucose-stimulated insulin secretion in InsGLuc-MIN6 cells after 24 h treatment with DMSO (0.2%), SW (5 μM), dextrorphan (DXO; 10 μM), or glimepiride (10 μM) in the presence or absence of nifedipine (10 μM). Data are the mean ± SD of *n* = 8. *p < 0.05 by two-way ANOVA with Dunnett’s multiple comparisons test. (B) InsGLuc-MIN6 cells treated as in (A), except testing different structurally distinct hypersecretion-inducing compounds (5 μM) and Tg (100 nM). Data are the mean ± SD of *n* = 3. *p < 0.05 by two-way ANOVA with Dunnett’s multiple comparisons test. See also Figure S3.

Many physiological and pharmacological stimuli can enhance Ca^2 +^ influx and potentially cause hypersecretion, likely through multiple mechanisms, including VDAC1. We have shown previously that ≤2 h exposure to SW016789 potentiated nutrient-induced Ca^2+^ influx to cause insulin hypersecretion. ^24^ However, ≥4 h of exposure significantly inhibited glucose-stimulated insulin secretion (GSIS) responses without causing cell death. We hypothesized that the effects of SW016789 may not be unique, as other pharmacological stimulators of insulin secretion have been shown to lead to suppression of β cell function, as is the case for the NMDA receptor antagonist dextrorphan (DXO)^26^ and sulfonylureas.^21,24,26,27,31^ Consistently, we observed a loss of function in MIN6 cells treated chronically with DXO or the sulfonylurea glimepiride (Figure 3A). We also discovered structurally distinct small-molecule hypersecretion inducers in a previous high-throughput screen (Figure S3A).^32^ These compounds had activity similar to SW016789, with 24 h pre-treatment leading to suppressed Ca^2+^ influx (Figures S3B–S3D) and insulin secretion responses (Figure 3B) in MIN6 cells. Preventing Ca^2+^ influx using the VDCC blocker nifedipine conferred protection from the chronic inhibitory effects of many of these hypersecretion inducers (Figure 3A and 3B). Nifedipine did not protect against thapsigargin-induced loss of function, distinguishing the hypersecretory response from ER stress. We also tested for potential effects of SW016789 *in vivo* on glucose tolerance. However, after an acute 5 mg/kg dose, there was no impact on glucose tolerance (Figures S3E). Considering the distal effects of hypersecretion on β cell function, from here we used SW016789 as an *in vitro* tool to induce hypersecretion to investigate the downstream dynamics of this response in β cells and to compare with ER stress responses.

### Identification and analysis of time-dependent transcriptomic changes in response to hypersecretory stress in β cells

**Figure 4.**
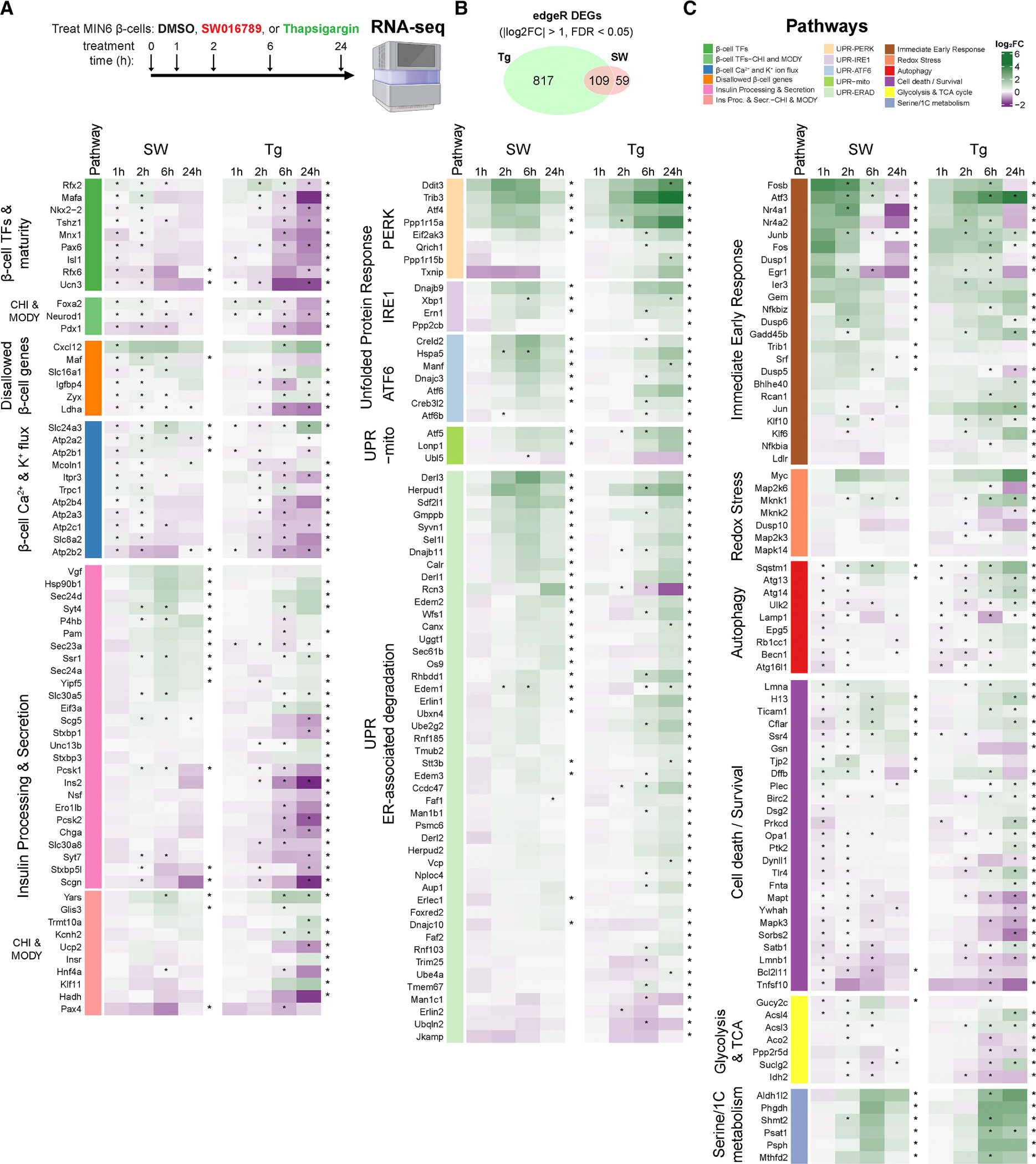
Time-course temporal transcriptomics uncovers differential effects of hypersecretion and ER stress (A) MIN6 β cells treated with DMSO (0.1%), SW (5 μM), or Tg (100 nM) for 1, 2, 6, and 24 h. *n* = 3. (B) Time-course edgeR analysis results showing overlap of differentially expressed genes (DEGs) between Tg and SW. (C) Heatmaps showing selected pathways containing DEGs that were significant in SW, Tg, or both. Asterisks inside heatmap cells indicate FDR < 0.05 for treatment vs. DMSO for that time point. Asterisks to the right of each gene row indicate significance (FDR < 0.05) across the entire time course vs. baseline. See also Figure S4.

**Figure 5.**
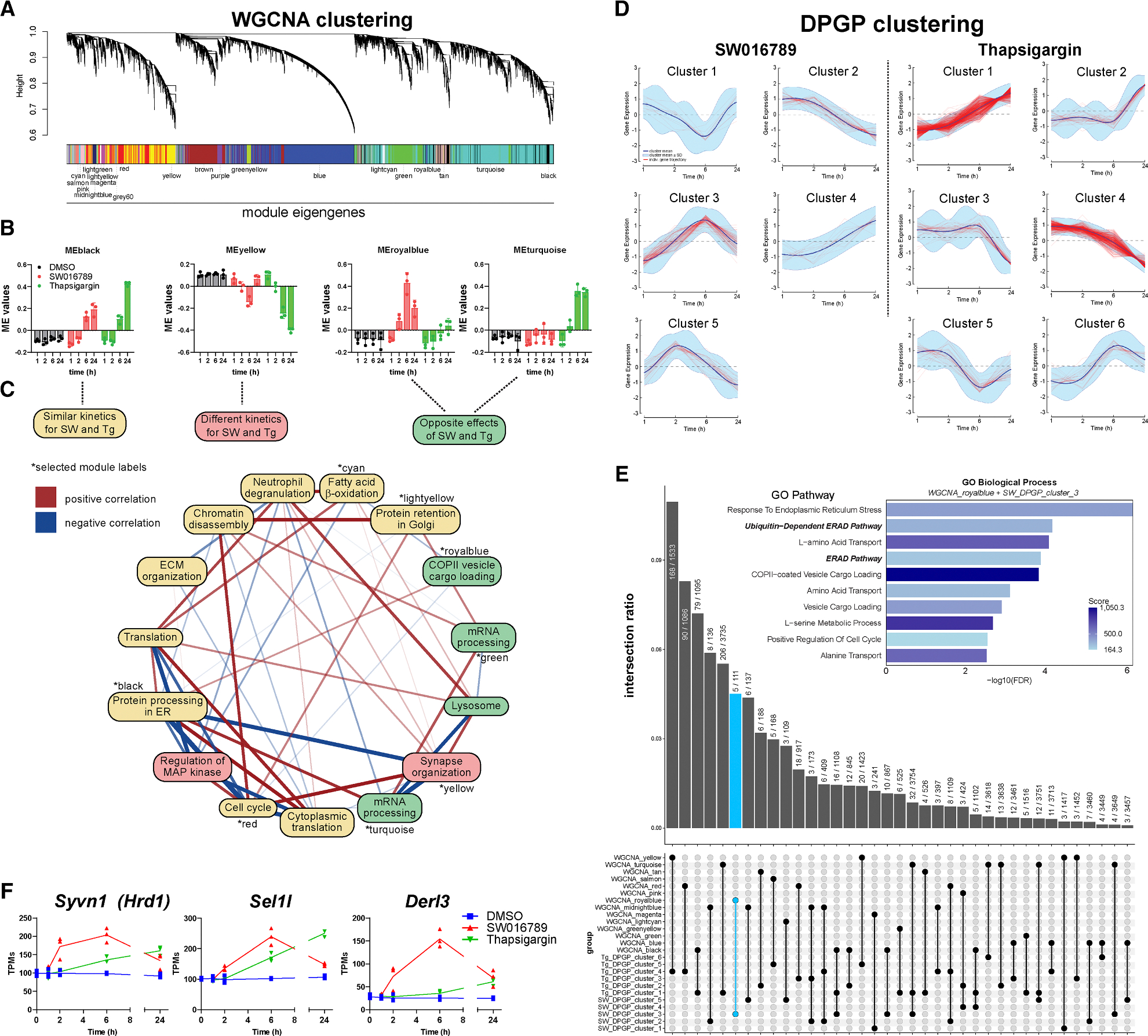
Clustering analyses unveil a distinct β cell hypersecretory response signature (A) Weighted gene co-expression network analysis (WGCNA) of the unfiltered transcriptomics dataset. (B) Module eigengene (ME) values for modules that have similar kinetics between SW and Tg (black), different kinetics (yellow), or opposite directional effects (royalblue and turquoise). Data are the mean ± SD of *n* = 3. (C) Directed network analysis of MEs with their top enriched ontology shown and colored by their kinetics and effect directions. Edges connecting the nodes are colored by positive (red) or negative (blue) correlation, and thickness indicates the magnitude of the correlation, with thicker edges showing stronger relationships. (D) Dirichlet process-Gaussian process (DPGP) clustering analysis. (E) UpSet plot comparison of all WGCNA modules and DPGP clusters. The overlap between SW_DPGP_cluster_3 and the royalblue module is highlighted in light blue. These two sets were merged and analyzed by GO biological process (inset). (F) Select genes for the ERAD pathway show differential expression dynamics between SW and Tg. Values shown are transcripts per million (TPMs) from RNA-seq data (*N* = 3). See also Figure S5.

To identify transcriptomic alterations downstream of hypersecretory stress, MIN6 β cells were treated with DMSO, SW016789, and thapsigargin for 1, 2, 6, and 24 h and submitted for RNA sequencing (RNA-seq) (Figure 4A; Table S2). Time-course differentially expressed gene (DEG) analysis (|log_2_ Foldchange| > 1 and false discovery rate [FDR] < 0.05) indicated 926 genes altered by thapsigargin and 168 genes altered by SW016789 with 109 in common (Figure 4B). We noted distinct expression differences between SW016789 and thapsigargin for multiple pathways (Figure 4C). For example, thapsigargin altered genes involved in the UPR^ER^, ERAD, redox stress, autophagy, cell death, insulin processing and secretion, disallowed β cell genes, metabolism, and β cell transcription factors. SW016789 selectively impacted subsets of the UPR^ER^, immediate-early response, and insulin secretion genes. We also compared SW016789-induced genes to published RNA-seq datasets of mouse islets treated with the NMDA receptor antagonist DXO (Figures S4A and S4B), ^26^ islets from mice chronically treated with glibenclamide (Figures S4C), ^33^ and fluorescence-activated cell sorting-purified β cells from *Abcc8* knockout mice (Figure S4D). ^34^ We found overlapping DEGs between SW016789 and DXO, including immediate-early genes and ERAD components (Figures S4A and S4B; Table S3). We also found that SW016789 induced genes in the serine biosynthetic pathway (Figure 4D), including *Psat1*, which was also increased in *Abcc8* knockout β cells (Figure S4D; Table S3). This finding agrees with data showing elevated serine biosynthesis in islets from patients with congenital hyperinsulinism. ^35^ There was limited overlap between the effects of SW016789 and chronic glibenclamide (*Angptl6*, *Gc*, *Nnat*, *Ppargc1a*, and *Slc7a11*), potentially suggesting distinct pathway engagement (Figure S4C; Table S3). Notably, SW016789 did not alter certain maladaptive responses like *Txnip*, *Qrich1*, or cell death-related genes. However, many distinctions in gene expression between SW016789 and thapsigargin appeared to be related to their temporal expression pattern. Therefore, to better understand these dynamic expression changes, we clustered the data using two different approaches. We used weighted gene co-expression network analysis (WGCNA) to cluster all genes in the raw RNA-seq count matrix into modules based on their expression patterns regardless of significance or fold change in edgeR (Figure 5A and S5; Table S3). For example, the “royalblue” and “turquoise” WGCNA modules stood out as robustly changed in opposite directions by SW016789 and thapsigargin (Figure 5B). We performed gene set enrichment analysis on each module using EnrichR-KG and created a directed network comprised of all modules with significantly enriched terms (Figure 5C). This network illustrated the differences between hypersecretion and thapsigargin-mediated ER stress and showed positive correlations between WGCNA modules like royalblue, which was enriched for secretory pathway genes and “black,” which was enriched for protein processing in the ER.

In parallel, we used Dirichlet process-Gaussian process (DPGP) to cluster DEGs from Figure 4A (Figure 5D).^36^ Notably, most SW016789-altered genes fell into a single cluster (SW_DPGP_cluster_3), which followed a pattern of early downregulation followed by increased expression peaking at 6 h and returning to baseline by 24 h. We noted that SW_DPGP_cluster_2 and SW_DPGP_cluster_5 exhibited a pattern of peak expression at 1 and 2 h, respectively, likely due to the immediate-early response downstream of enhanced Ca^2+^ influx. For thapsigargin, most genes fell into Tg_DPGP_cluster_1 and Tg_DPGP_cluster_4, which represent genes that continuously increase or decrease with time, respectively. We compared the WGNCA and DPGP clustering results using an UpSet plot ranked by the intersection ratio (Figure 5E). This indicated that the royalblue WGCNA module was the most similar to SW_DPGP_cluster_3 but included additional genes that had not reached significance in edgeR analyses. SW_DPGP_cluster_3 also overlapped with Tg_DPGP_cluster_1, highlighting genes that are modulated transiently after hypersecretion but that are induced more slowly by ER stress. For our downstream analyses, we combined the gene lists of royalblue and SW_DPGP_cluster_3 because of their similar expression dynamics. We propose that this combined set represents a hypersecretion response signature. Within this signature, we found enrichment of pathways related to ERAD and ER stress (e.g., *Derl3*, *Syvn1*, *Sel1l*, *Hspa5*, and *Ppp1r15a*), amino acid transport, vesicle trafficking (e.g., the COP-II complex), and serine metabolism (e.g., *Psat1*, *Psph*, and *Phgdh*) (Figure 5E, inset). ERAD has been implicated as an important pathway in β cell function, ^37–40^ so we hypothesized that it could serve an adaptive role during hypersecretion. ERAD mediates the extrusion and ubiquitination of misfolded proteins from the ER membrane and lumen and thereby mitigates ER stress and eventual apoptosis. ERAD factors like *Sel1l*, *Derl3*, and *Syvn1* are induced by SW016789 at 6 h, while their induction by thapsigargin is either weaker or delayed (Figure 5F). We next investigated the involvement of ERAD in the β cell hypersecretion response and diabetes pathology using pharmacological treatments, immunoblotting in murine and human β cell lines, and immunostaining of human pancreas sections.

### ERAD activity may be altered by hypersecretion and may protect against apoptosis

**Figure 6.**
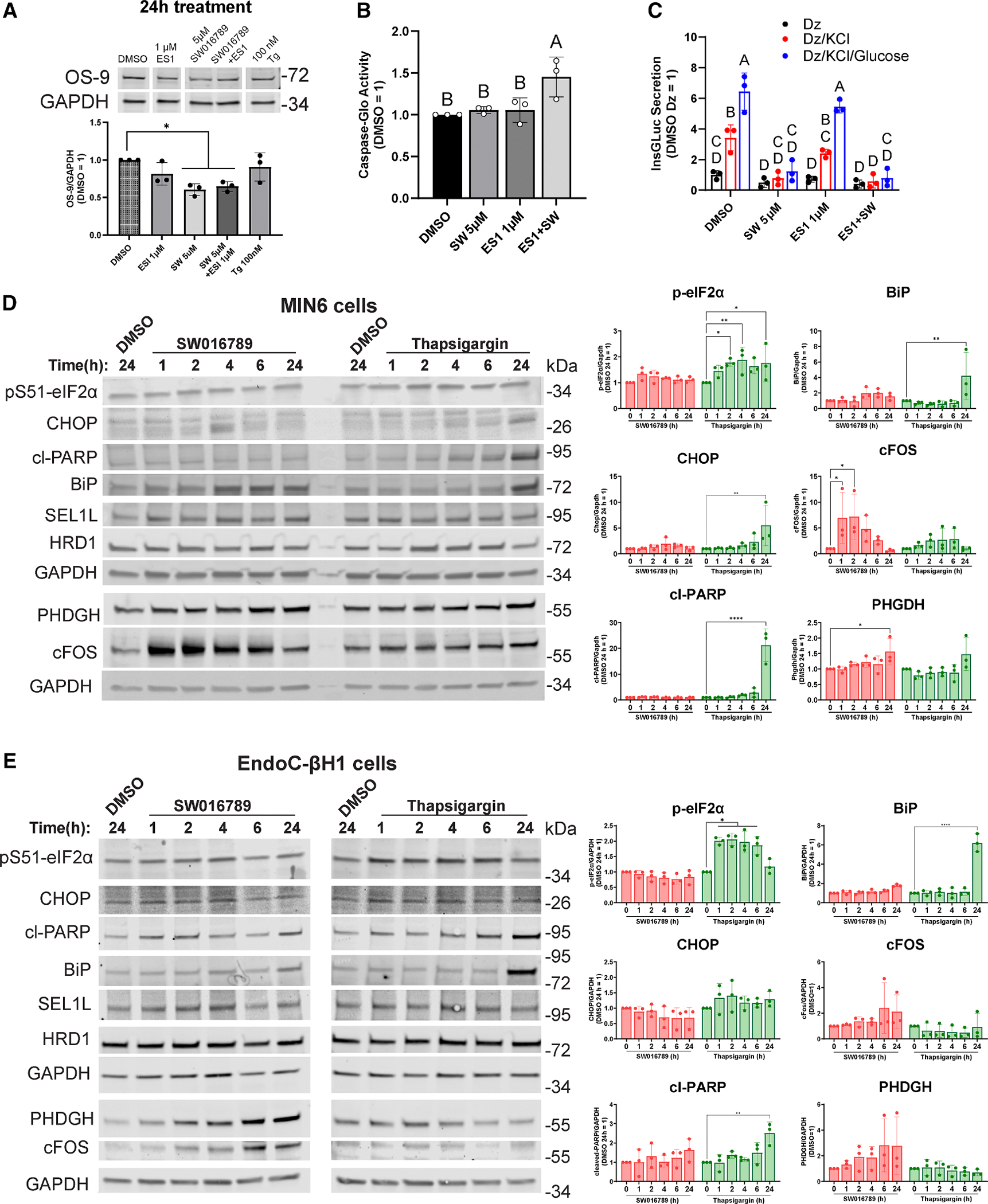
Effects of hypersecretion and ER stress on the expression of ERAD, ER stress, and immediate-early response proteins (A) OS-9 immunoblots from MIN6 cells treated for 24 h with 1 μM eeyarestatin I (ES1), 5 μM SW, ES1 + SW, or 100 nM Tg. Data are the mean ± SD of *n* = 3. *p < 0.05 by one-way ANOVA with Dunnett’s multiple comparisons test. (B) Caspase-Glo assay in MIN6 cells exposed to the indicated treatments for 24 h. Data are the mean ± SD of *n* = 3. Statistical differences (*p* < 0.05 by one-way ANOVA with Tukey’s multiple comparisons test) are labeled by different lettering. (C) InsGLuc-MIN6 secretion assay in cells treated for 24 h with the indicated drugs, followed by stimulating using diazoxide (250 μM), KCl (35 mM), and glucose (20 mM). Data are the mean ± SD of *n* = 3. Differences (*p* < 0.05 by two-way ANOVA with Tukey’s multiple comparisons test) are labeled by different lettering. (D) MIN6 β cells were treated with SW (5 μM), Tg (100 nM), or DMSO (0.1%) for the indicated times, and samples were analyzed by immunoblotting. Data are the mean ± SD of *n* = 3. *p < 0.05 vs. respective DMSO control by two-way ANOVA with Dunnett’s multiple comparisons test. (E) Human EndoC-βH1 cells were treated as in (D), except Tg was used at 1 μM. Data are the mean ± SD of *n* = 3. *p < 0.05 vs. respective DMSO control by two-way ANOVA with Dunnett’s multiple comparisons test. Vertical white spaces between blots in (A) and (E) indicate that superfluous lanes were eliminated. See also Figure S6.

As one readout of ERAD activity, we monitored the abundance of the ERAD substrate OS-9. ^41^ We treated β cells with Tg, SW016789, and eeyarestatin I (ES1), which inhibits VCP/p97, the enzyme responsible for peptide extrusion through the ERAD complex. SW016789 alone or in combination with ES1 reduced OS-9 abundance (Figure 6A), potentially reflecting increased ERAD flux. It is worth noting that OS-9 mRNA expression is increased by both SW016789 and Tg at 24 h (Figure 4C). ERAD may contribute to β cell survival during the hypersecretory response, while Tg-treated cells undergo chronic ER stress and apoptosis. To test this, we exposed β cells to either ES1 or SW016789 alone or in combination for 24 h. Only the combination of ES1 and SW016789 increased caspase-3/7 activity (Figure 6B), suggesting a requirement for ERAD activity for β cell survival during hypersecretion. MIN6 β cells retained glucose responsiveness after 24 h treatment with ES1, indicating that ERAD inhibition under these conditions did not reduce β cell function (Figure 6C). As expected, SW016789 alone or in combination with ES1 caused loss of function (Figure 6C).

### Hypersecretion responses at the protein level induce the immediate-early response and do not robustly engage canonical ER stress pathways

We next determined the extent to which transcriptional alterations due to hypersecretion or canonical ER stress led to changes in protein abundance. Mouse MIN6 cells and human EndoC-βH1 cells were treated with SW016789 or Tg for 1, 2, 4, 6, and 24 h as well as the vehicle control DMSO for 24 h. In MIN6 β cells treated with Tg, we observed significantly increased amounts of phosphorylated eIF2α by 2 h, followed by elevated CHOP, cleavage of poly(ADP-ribose) polymerase (PARP), and BiP by 24 h (Figure 6D). However, with SW016789-induced hypersecretion, we observed a rapid elevation of c-Fos, followed by increased PHGDH expression at 24 h, with minimal impact on eIF2α phosphorylation, CHOP, BiP, or the ERAD-related proteins SEL1L, HRD1 (also known as SYVN1), DNAJC3, and DNAJB11 (Figures 6D and S6A). These findings were largely mirrored in human EndoC-βH1 cells (Figures 6E and S6B), although the immediate-early response, as measured by c-Fos expression, was less pronounced in EndoC-βH1 than in MIN6 β cells. Overall, these findings support a conserved response to hypersecretion compared to ER stress in mouse and human β cells. Not all transcriptional differences are reflected in steady-state protein abundance, or the magnitude of the alterations is not large enough to reach significance, as seen for CHOP, DNAJC3, and DNAJB11 in MIN6 cells (Figure 6D) or c-Fos, phosphoglycerate dehydrogenase (PHGDH), and DNAJB11 in EndoC-βH1 cells (Figures 6E and S6B).

### The ERAD components HRD1, SEL1L, and DERL3 are co-expressed in human islets from normal and T2D donors

**Figure 7.**
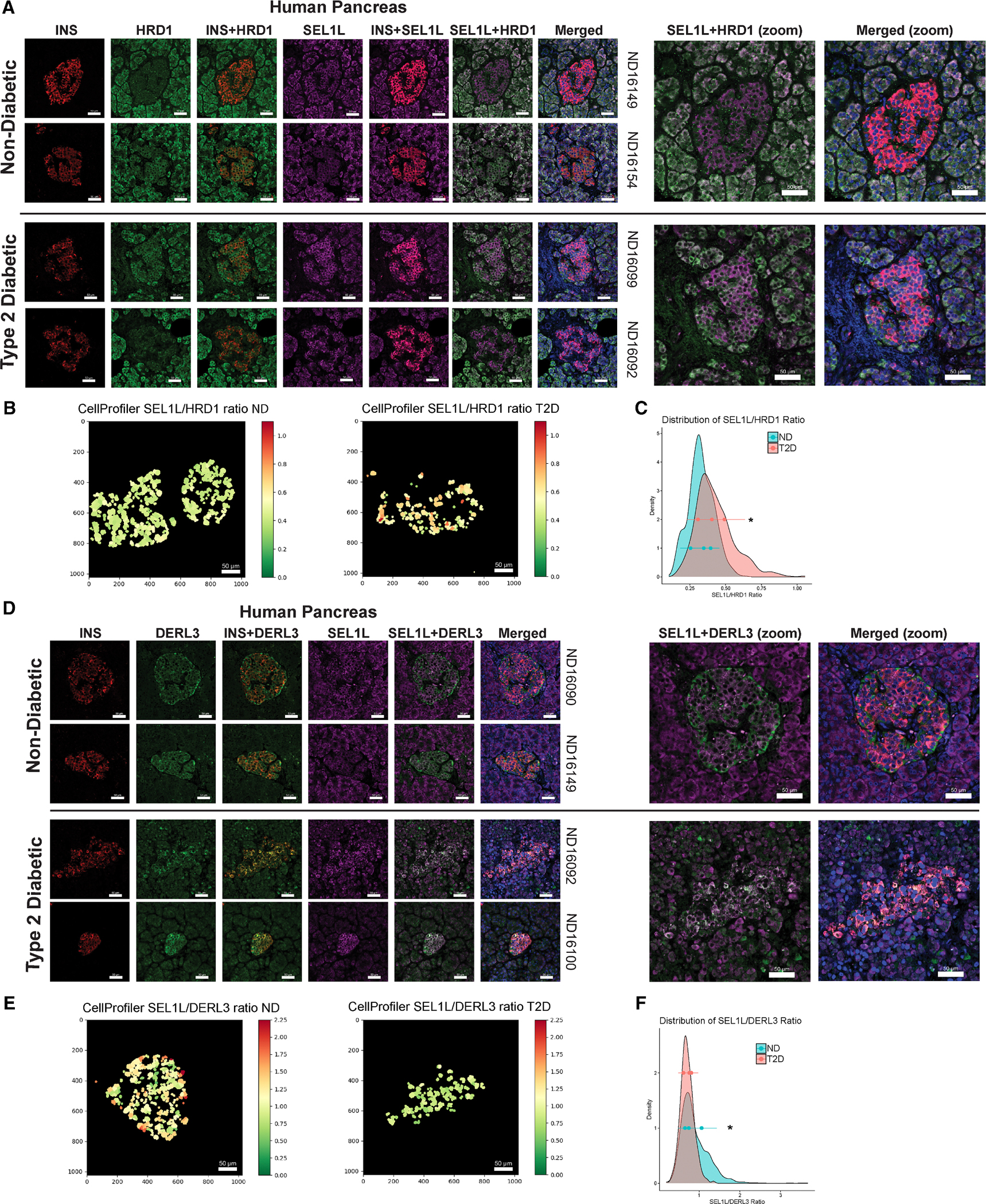
Expression of ERAD components in non-diabetic and T2D human pancreas (A) Immunohistochemical staining of insulin (INS; red), HRD1 (green), and SEL1L (violet) and overlaid images showing INS+HRD1, INS+SEL1L, and HRD1+SEL1L. Donor identifiers are shown on the right. (B) CellProfiler quantification of the ratio of β cell intensities for SEL1L and HRD1. Axes are pixels (1,024 × 1,024), 120 pixels = 50 μm (C) Histogram plot of all quantified β cell SEL1L/HRD1 ratios. *p < 0.05 by Kolmogorov-Smirnov test. (D) Immunohistochemical staining for INS (red), DERL3 (green), and SEL1L (violet) and overlaid images showing INS+DERL3 and SEL1L+DERL3. All images shown are representative of three independent non-diabetic (ND) and T2D donors. (E) CellProfiler quantification of the ratio of β cell intensities for SEL1L and DERL3. *p < 0.05 by Kolmogorov-Smirnov test. (F) Histogram plot of all quantified β cell SEL1L/DERL3 ratios. Histogram plots in (C) and (F) also show the mean ± SD of *n* = 3 donors (3–5 imaged regions per donor). All scale bars represent 50 μm. See also Figure S7.

In both non-diabetic and T2D pancreas tissue, HRD1 and SEL1L staining appeared across both endocrine and exocrine cells (Figures 7A, S7C, and S7D). Within islets, HRD1 and SEL1L staining overlapped with insulin staining, indicating expression in β cells. SEL1L and HRD1 were also present in islets from T2D donors, although the islet structure appeared more disrupted in T2D compared to non-diabetic samples. HRD1 and SEL1L colocalized in islets, but the relative amount of SEL1L compared to HRD1 appeared to be higher in T2D islets. We reasoned that alterations in the stoichiometry of ERAD components could contribute to β cell dysfunction in T2D. Using CellProfiler, we analyzed the ratio of SEL1L/HRD1 intensity within each β cell and plotted the distribution of the SEL1L/HRD1 ratio (Figure 7B). Q-Q plots indicated that the ratios did not follow a normal distribution (Figures S7A and S7B). The distribution of SEL1/HRD1 ratios was significantly right-shifted in T2D (Figure 7C). Because the main ERAD complex contains HRD1, SEL1L, as well as DERL3, whose induction is generally more responsive to stress conditions, ^42^ we also stained DERL3 in the same human samples (Figure 7D and S7E). DERL3 staining was enriched in islets compared to surrounding acinar tissue and, in T2D, appeared to colocalize more with β cells. DERL3 and SEL1L colocalization in islets appeared more prominent in T2D than in non-diabetic samples. Like with SEL1L/HRD1, we measured the distribution of the SEL1L/DERL3 ratio in β cells and found that it was significantly left-shifted in T2D (Figure 7E and 7F). Taken together, these data suggest that at least a subset of T2D β cells has lower HRD1 and higher DERL3 abundance relative to SEL1L. These data also confirm the co-expression HRD1, SEL1L, and DERL3 at the protein level in non-diabetic and T2D human islets. The presence of these components in T2D β cells suggests that it may be possible to target ERAD flux to ameliorate β cell stress in diabetes.

## DISCUSSION

### The role of Vdac1 in SW016789-mediated β cell hypersecretion

VDACs comprise three isoforms encoded by *VDAC1*, *VDAC2*, and *VDAC3*. The VDACs are permeable to ions (K ^+^, Na^+^, and Ca^2+^ and metabolites, are expressed in β cells, ^29,43^ and have been pursued as possible therapeutic targets for diabetes. ^44^ VDAC2 has been suggested to protect against cell death by sequestering the pro-apoptotic protein Bak.^45^ VDAC3 may play a larger role in testis tissue and have functions distinct from VDAC1 and VDAC2. ^46^ However, VDAC1 expression has been linked to T2D and β cell dysfunction. ^29^ VDAC1 was mistargeted to the plasma membrane, where it co-localized with soluble NSF-attachment receptor (SNARE) proteins, and has been suggested to impair GSIS in the setting of glucotoxicity and T2D. ^29^ It is worth noting that mitochondrial VDAC1 protein is reduced in T2D β cells. ^47^ VDAC1 has been suggested to interact with β cell glucokinase, ^48^ which could underlie its role in dysfunctional β cells in T2D. ^29^ VDAC1 could also be involved in mitochondrion-ER contact sites, as it interacts with IP3R2, and this interaction is reduced in T2D β cells. ^47,49^

Our data support that SW016789 acts through VDAC1. However, SW016789 may exhibit polypharmacology and act through additional targets. Based on our current findings and previous phenotypic characterization, ^24^ we propose that SW016789 acts at the plasma membrane level to influence Ca^2+^ influx through VDCCs. It is possible that some effects of SW016789 are modulated by phosphorylation of VDCCs or other ion channels that indirectly potentiate VDCC activation. ^50^ While VDAC1 is purported to be present in both the mitochondrial outer membrane and the plasma membrane, our prior work did not uncover mitochondrial alterations. ^24^ While we have not specifically investigated plasmalemmal versus mitochondrial VDAC1, we predict that SW016789 is acting through plasma membrane-localized VDAC1. These findings are relevant to the pursuit of VDAC1 as a target in T2D and Alzheimer’s disease. ^29,51,52^

### β cell adaptation to the spectrum of secretory stresses

Hypersecretion induces a distinct response compared to pharmacological or genetic inhibition of SERCA2, a conclusion supported by our findings and others. ^53^ Hypersecretory stress is likely different than ER stress that occurs in response to the N-linked glycosylation inhibitor tunicamycin, glucolipotoxicity, antioxidants like dithiothreitol, or induction of misfolding mutants of insulin (i.e., Ins2-C96Y, Akita mice). ^54,55^ A major distinction is that, in response to hypersecretion *in vitro*, β cells can sacrifice their function to stave off death, which they cannot do in response to many other stressors. There is evidence from genetic mutations in humans (KCNQ1 ^56^ and HNF1A ^18)^ and mice (SUR1 ^57)^ that hypersecretion precedes β cell failure. Pharmacological evidence suggests that chronic sulfonylurea use can be detrimental to β cell function. ^21,31,58–62^ However, the transcriptomic responses to hypersecretion induced by different small molecules may be distinct, as we observed for SW016789 compared to glibenclamide (Figure S4C). β cells may handle hypersecretion largely by downregulating insulin secretion, but over time, this adaptive strategy becomes maladaptive *in vivo*. Reduced insulin secretion results in hyperglycemia, which contributes to β cell failure in a vicious cycle. ^25^ How β cells achieve a suppressed secretory state and the extent to which specific genes may protect β cells from death in hypersecretion is an active area of research. We identified multiple clusters of dynamic gene expression in our analysis that will require further investigation. Clusters containing immediate-early response genes, or those specifically induced by thapsigargin and not hypersecretion, may contain cell viability regulators. For example, *Npas4* was expressed among the immediate-early genes, and this gene has been demonstrated to have a pro-survival role in the β cell. ^63,64^

Other recent findings support the idea that β cell survival under hypersecretory conditions can occur at the expense of secretory function; for example, by treating mouse islets with DXO. ^26^ This effect has been suggested to depend on ATF4-mediated expression of serine-linked mitochondrial one-carbon metabolism, involving the genes *Phgdh*, *Shmt2*, and *Mthfd2*. Even though we analyzed different time points, we also found genes in this pathway induced by hypersecretion (SW_DPGP_cluster_3). Our findings support the possibility that the effects of SW016789-induced hypersecretion may also involve serine and one-carbon metabolism in β cells to protect against stress-induced cell death. It is possible that hypersecretion induced by a variety of pharmacological avenues can lead to similar outcomes in the β cell, likely via converging on enhanced Ca^2+^ influx.

### Blocking Ca^2+^ influx to protect against hypersecretory stress

We have observed previously that nifedipine prevents SW016789-induced hypersecretory stress and loss of function in β cells. ^24^ Furthermore, nifedipine acted similarly for many small-molecule secretion enhancers we identified in our high-throughput screen, including sulfonylureas. This indicates that, while it may be useful to understand the proximal mechanisms causing hypersecretion, Ca^2+^ influx is a key factor. Interestingly, not all inhibitors of insulin secretion confer the same protection as nifedipine. SW016789, in the presence of glucose, elicited Ca^2+^ influx and insulin release even when treated with the K_ATP_ channel opener diazoxide. ^24^ Additionally, dihydropyridine-based VDCC blockers (such as nifedipine) are superior in protecting against SW016789 compared to verapamil or diltiazem. ^24^ Data showing that VDCC activators also induce hypersecretion and phenocopy SW016789 point to the importance of Ca^2+^ influx in this distinct stress response. ^24,27^ The different mechanisms of action of VDCC modulators may explain why nifedipine protects against hypersecretory stress. Diltiazem and verapamil block ion permeation through the VDCC pore, while nifedipine interacts with a different site near the pore without blocking it.^65,66^ Dihydropyridines may selectively inhibit VDCCs under the chronic depolarization that occurs during hypersecretion. ^66,67^

### β cell ERAD may be a protective mechanism upregulated during hypersecretion

Among genes in SW_DPGP_cluster_3, there was an enrichment of the core ERAD components *Sel1l*, *Syvn1* (HRD1), and *Derl3*. Previous work supports a requirement for well-regulated ERAD in β cell function. HRD1 is upregulated in islets of Akita mice, which become diabetic because of β cell ER stress from a misfolding insulin mutant. ^68^ HRD1 has also been found to be increased in T2D human islet β cells, and β cell-specific HRD1 overexpression in mice impairs GSIS.^69^ This loss of β cell function may be due to HRD1-mediated degradation of the critical transcription factor MafA. However, SW016789 did not alter MafA protein levels, ^24^ nor did it substantially alter transcript levels of β cell transcription factors. Conversely, loss of SEL1L in mouse β cells led to impaired function, loss of β cell identity, and development of diabetes. ^39,40^ These findings agreed with a loss of SEL1L in β cells from human T2D donors. ^39^ For the third core ERAD components, DERL1/2/3, not much is known about their roles in β cells. Combined, these published findings suggest both positive and negative roles for ERAD in β cell function and T2D pathogenesis. Our immunostaining in human β cells from normal and T2D donors suggests an imbalance in the relative abundance of HRD1 and DERL3 compared to SEL1L. Different stoichiometries between these components may indicate the formation of distinct ERAD complexes with different substrates or throughput. Indeed, altered levels of HRD1 can affect ERAD complex stoichiometries, ^70^ and distinct ERAD complexes containing either DERL1 or DERL3 have been described. ^42^ DERL3 may also act as a hub molecule to control the formation of different ERAD complexes. For example, DERL3 can interact with another ERAD component, homocysteine inducible ER protein with ubiquitin like domain 1 (HERP1), and in pancreata of *Derl3* knockout mice, the expression of DERL1 and DERL2 was reduced in a HERP1-dependent manner. ^71^ More detailed analyses and tool development are needed for investigating ERAD flux in non-diabetic vs. T2D β cells and for understanding how hypersecretion may influence this process.

### Limitations of the study

In this work, we described the identification of VDAC1 as a protein target for the small-molecule hypersecretion inducer SW016789. While this is supported by affinity purification-coupled proteomics, CETSA, RNA interference, and pharmacological experiments, it is possible that SW016789 also acts through other target proteins in the β cell. We also provided a transcriptomic resource for the time course of differential gene expression during hypersecretion induced by SW016789 or ER stress induced by Tg. While these data were generated using the MIN6 mouse β cell line, validation was performed in human EndoC-βH1 cells to show that human cells exhibit a similar response (Figure 1A; 6E). Although we did not confirm induction of specific proteins in human islets, we showed that some of the same genes are induced by SW016789 at the mRNA level in primary human islets of both male and female donors (Figure 1C). This agrees with our previous finding that SW016789 causes human islets to lose GSIS responses. ^24^ Additionally, the potential *in vivo* impacts of SW016789 have not been determined. However, we used SW016789 as an *in vitro* tool compound for this study, not as a candidate therapeutic agent. *In vivo* characterization would require extensive pharmacokinetic and pharmacodynamic studies that are beyond the scope of the current work. Bulk RNA-seq experiments, while having the advantage of improved read depth, lack the ability to detect heterogeneity between individual β cells during these stress responses. Single-cell transcriptomic experiments are required to better define and understand the contributions of β cell subpopulations in hypersecretion and during diabetes pathogenesis, an issue recent studies have begun to address. ^72^ Finally, the expression of ERAD components was implicated in our transcriptomic studies using SW016789. While OS-9 levels were altered, we have not exhausted all potential readouts for ERAD flux in this work. The p97/VCP inhibitor ES1 may have off-target effects, ^73,74^ and inducible β cell knockdown/knockout of ERAD components will be ideal in the future for addressing their role(s) in adaptation to hypersecretory stress. In conclusion, further studies are needed to improve our understanding of hypersecretion downstream of enhanced Ca^2+^ influx and exocytosis and thus reveal potentially targetable components for treating diabetes or other β cell diseases.

## RESOURCE AVAILABILITY

### Lead contact

Requests for further information and resources should be directed to and will be fulfilled by the lead contact, Michael A. Kalwat (mkalwat@indianabiosciences.org).

### Materials availability

Making the Z629 probe available is not feasible because of the limited shelf life. Resynthesis is possible following the procedures in the manuscript. No other unique reagents were generated in this work.

### Data and code availability

- The datasets generated for this study can be found in the NCBI GEO: GSE194200 and ProteomeXchange: PXD050590. Other datasets used for comparisons are available at NCBI GEO: GSE238017, ArrayExpress: E-MTAB-4726, Mendeley Data: https://doi.org/10.17632/g4bdvw6czr.2, and Figshare: https://doi.org/10.2337/figshare.28003664).
- Code is publicly available for time-course transcriptomics analysis at Github: https://github.com/kalwatlab/hypersecretion-timecourse and for DPGP at Github: https://github.com/PrincetonUniversity/DP_GP_cluster.
- Any additional information required to reanalyze the data reported in this paper is available from the lead contact upon request.

## STAR★METHODS

### EXPERIMENTAL MODEL AND STUDY PARTICIPANT DETAILS

#### β-cell line culture

Culture of MIN6 β-cells has been described. ^91^ MIN6 cells were authenticated using the Mouse STR cell line validation kit from ATCC (#137-XV). MIN6 cells were cultured in high glucose DMEM containing 10% FBS, 50 μM beta-mercaptoethanol, 1 mM pyruvate, 292 μg/mL L-glutamine, 100 U/ml penicillin, and 100 μg/mL streptomycin. InsGLuc-MIN6 cells were generated using the MIN6 cell line and characterized as previously described and were determined to be negative for mycoplasma. ^91^ InsGLuc-MIN6 were cultured as described for MIN6 cells above, with the addition of 250 μg/mL G418 to the medium. MIN6 β cells were treated with DMSO, SW016789 and thapsigargin for 1, 2, 6 and 24 h and RNA was collected for each time point at the end of the time course. EndoC-βH1 cells were from Human Cell Design. EndoC-βH1 cells were authenticated using the Human STR cell line validation kit from ATCC (#135-XV). Culture of EndoC-βH1 cells has been described. ^92^ Briefly, EndoC-βH1 cells were cultured in low glucose DMEM containing 2% fatty acid-free bovine serum albumin (BSA), 50 μM 2-β-mercaptoethanol, 10 mM nicotinamide, 5.5 μg/mL transferrin, 6.7 ng/mL sodium selenite, 4 mM L-glutamine, 1mM pyruvate, 100 U/ml penicillin, and 100 μg/mL streptomycin. The cells were seeded on plates coated with 1.2% Matrigel containing plus 3 μg/mL fibronectin and cultured at 37 °C and 5% CO_2_. Cell lines were determined to be negative for mycoplasma.

#### Human islet culture

Cadaveric human islets were obtained through the Integrated Islet Distribution Program (IIDP) and Prodo Labs. Islets were isolated by the affiliated islet isolation center and cultured in PIM medium (PIM-R001GMP, Prodo Labs) supplemented with glutamine/glutathione (PIM-G001GMP, Prodo Labs), 5% Human AB serum (100512, Gemini Bio Products), and ciprofloxacin (61–277RG, Cellgro, Inc) at 37 °C and 5% CO2 until shipping at 4 °C overnight. Human islets were cultured upon receipt in complete CMRL-1066 (containing 1 g/L (5.5 mM) glucose, 10% FBS, 100 U/ml penicillin, 100 μg/mL streptomycin, 292 μg/mL L-glutamine). Experiments included both male (*n* = 3) and female (*n* = 2) donors. Due to sample size, data were not stratified by sex. Human islet information and donor metadata are provided in Table S6.

#### Human pancreas tissue

Formalin-fixed paraffin-embedded (FFPE) de-identified human pancreas tissue were obtained through the National Disease Research Interchange (NDRI) (*n* = 4 non-diabetic, *n* = 3 T2D). Results from male and female donors were included in the study, however only male T2D samples were available. Donor characteristics and metadata are provided in Table S5.

#### Mice

Twelve healthy wild-type male C57BL6/J mice (8–9 weeks old) from three different litters were from the breeding colony at UW Madison Biochemistry. Mice were bred by sibling mating. Mice were less than five generations removed from Jackson Laboratories stock. Mice were housed at 2–4 per cage with *ad libitum* food and water access, with a 12 h light/dark cycle. Mice were not part of any previous procedures.

### METHOD DETAILS

#### Secreted insulin-Gaussia luciferase assays

InsGLuc secretion assays were performed as previously described. ^91,93^ Briefly, InsGLuc-MIN6 cells were plated in 96-well dishes at 1e5 cells/well and cultured for 3–4 days before compound treatment either overnight (24 h) in medium or acutely (1 h) in freshly prepared glucose-free modified Krebs-Ringer bicarbonate (KRBH) buffer (5 mM KCl, 120 mM NaCl, 15 mM HEPES, pH 7.4, 24 mM NaHCO_3_, 1 mM MgCl_2_, 2 mM CaCl_2_, and 1 mg/mL radioimmunoassay-grade BSA). Treatments in 96-well assays were performed with at least three replicate wells and in at least three independent passages of cells. For assays, cells were washed twice with KRBH and preincubated in 100 μL of KRBH (250 μM diazoxide where indicated) for 1 h. The solution was then removed, and cells were washed once with 100 μL of KRBH and then incubated in KRBH with or without the indicated stimulation and compound treatments for 1 h. 50 μL of KRBH was collected from each well and pipetted into a white opaque 96-well assay plates. Fresh GLuc assay working solution was then prepared by adding coelenterazine stock solution into assay buffer to a final concentration of 10 μM. 50 μL of working solution was then rapidly added to the wells using an Integra Voyager 1250 μL electric multi-channel pipette for a final concentration of 5 μM coelenterazine. Plates were spun briefly, and luminescence was measured on a Synergy H1M2 plate reader (BioTek). The Gen5 software protocol was set to shake the plate orbitally for 3 s and then read the luminescence of each well (integration, 100 ms; gain,150). High-throughput screening of compounds in the InsGLuc-MIN6 β-cell line was previously described. ^24,32^

#### Synthesis of Z629, photoaffinity analog of SW016789

The SW016789 analog, Z6292276622 (Z629), containing a fluorinated aryl azide and an alkyne-containing phenylacetylene, was synthesized by Enamine according to the scheme shown in Figure S1A. Step A: To a solution of 2,3,4,5,6-pentafluorobenzaldehyde (13.1 g; 0.067 mol) in acetone/water (100/50 mL), sodium azide (4.57 g; 0.7 mol; 1.05 eq) was added in one portion, and the mixture was stirred at 50 °C for 16 h. The mixture was then cooled to room temperature (RT) and concentrated *in vacuo* below 35 °C to obtain a ~60 mL solution. The resulting mixture was diluted with 200 mL of t-BuOMe, and the organic phase was separated and washed twice with brine, dried and concentrated to obtain crude 4-azido-2,3,5,6-tetrafluorobenzaldehyde (13 g; 0.0593 mol, Yield = 88%, 80% purity by NMR), which was used in the next step without further purification. Step B: NaBH4 (2.69 g; 0.071 mol; 1.2 eq) was suspended in THF (50 mL) and the mixture cooled to −20 °C under argon atmosphere (Ar). Dimethylamine hydrochloride (5.8 g; 0.071 mol; 1.2 eq) was added portion-wise at −20 °C, and the mixture was stirred for 1 h at this temperature and then 16 h at RT. The formed precipitate was filtered and the filtrate concentrated *in vacuo* to give dimethylamine borane. In a 500 mL flask 4-azido-2,3,5,6-tetrafluorobenzaldehyde (13 g; 0.059 mol; 1 eq) was dissolved in 100 mL of acetic acid, and the freshly prepared dimethylamine borane was added portion-wise at RT. The mixture was heated at 55 °C for 1hr and then concentrated *in vacuo* at 45 °C. The residue was dissolved in 200 mL of t-BuOMe, washed 4 times with 5% NaHCO_3_, dried (Na _2_SO_4)_, and concentrated *in vacuo*. The residue was purified by silica gel column chromatography (eluted by Hex:EtOAc 20:1 to 1:1) to obtain (4-azido-2,3,5,6-tetrafluorophenyl)methanol (7.5 g; 0.034 mol; yield = 57%) as pale yellow solid. Step C: A solution of (4-azido-2,3,5,6-tetrafluorophenyl) methanol (7.3 g 0.033 mol; 1 eq) in dry dichloromethane (100 mL) was cooled in an ice bath under Ar. Pyridine (2.8 mL; 0.034 mol; 1.05 eq) and PBr_3_ (1.25 mL; 0.013 mol; 0.4 eq) were added to this stirred solution via a syringe over a period of 30 min. After 16 h at RT, 2-propanol (80 mL) in dichloromethane (200 mL) was added, followed after 15 min by an equal volume of 5% NaHCO_3_. Extraction with dichloromethane and evaporation yielded a light-yellow solid that was triturated with t-BuOMe. The formed precipitate was filtered and washed by t-BuOMe. The filtrate was evaporated to obtain 1-azido-4-(bromomethyl)-2,3,5,6-tetrafluorobenzene (3.4 g; 0.0119mol; Yield = 36%) as a yellow solid. Step D: To a solution of 1-azido-4-(bromomethyl)-2,3,5,6-tetrafluorobenzene (2.6 g; 9.15 mmol; 1 eq) in 30 mL of DMF, 2-aminobenzimidazole (1.22 g; 9.15 mmol; 1 eq) was added, followed by the addition of K_2_ CO_3_ (2.02 g; 14.65 mmol; 1.6 eq). The mixture was stirred at room temperature for 16 h and then the mixture was poured into ice-water. The formed precipitate was filtered, washed with water, and concentrated *in vacuo* to obtain crude aminobenzimidazole **1** (2.2 g; 6.5 mmol, 60% purity by LCMS) as a light brown solid, which was used in the next step without further purification. Step E: The crude aminobenzimidazole **1** (2.2 g; 6.5 mmol, 60% purity by LCMS) and 2-bromo-1-(4-ethynylphenyl)ethan-1-one (1.59 g; 6.5 mmol; 1 eq) were dissolved in acetone (40 mL). Sodium iodide (1.03 g, 6.87 mmol; 1.05 eq) was added and the mixture was stirred at 50 °C for 19 h. The product was isolated as a solid via filtration and washed with acetone to obtain aminobenzimidazole **2** (0.8 g; 1.43 mmol; Yield = 15.6% for two steps) as an off-white solid. Step F: NaBH_4_ (0.024 g; 0.63 mmol; 1eq) was added portion-wise at −10 °C to a suspension of aminobenzimidazole **2** (0.35 g; 0.63 mmol; 1eq) in 10 mL of MeOH. The mixture was stirred for 30 min at room temperature and quenched with concentrated aqueous NH_4_ Cl. The mixture was concentrated *in vacuo* and diluted with EtOAc and water. The organic phase was separated, dried (Na_2_ SO_4)_, concentrated *in vacuo* and purified by HPLC (neutral phase) to obtain Z6292276622 (Z629) (0.012 g; 0.02 mmol; Yield = 4%) as an off-white solid.

#### Target deconvolution for SW016789

MIN6 β-cells and EndoC-βH1 cells were cultured in 10cm dishes to 80% confluence and then treated in complete medium with 5μM Z629 or DMSO as a control for 15 min in the dark at room temperature. Cells were exposed to UV-B light (306 nm) for 15 min on ice without lid. Cells were washed with cold PBS and lysed in RIPA buffer (50 mM HEPES pH 7.4, 0.5% sodium deoxycholate, 1% NP40, 150 mM NaCl, 0.1% SDS, and 25 U/mL benzonase). 1000 μg lysates per reaction condition were pre-cleared by rotating with high-capacity streptavidin-agarose beads for 1 h at room temperature. Click reaction was performed with the pre-cleared lysates with 0.0125 mM biotin-azide-plus, 1.25 mM sodium ascorbate, 0.05 mM THPTA, 1.25 mM CuSO_4_ for 15 min in the dark at room temperature. Proteins were then precipitated in 4 volumes of acetone, and samples were centrifuged at 20,000 × g for 10 min at 4°C to pellet insoluble proteins, then resolubilized in 4% SDS in PBS. The lysates were rotated overnight at 4 °C with high-capacity streptavidin-agarose beads in affinity purification buffer (50 mM HEPES, pH 7.4; 100 mM NaCl; 1% NP-40). For SDS-PAGE beads are washed twice with both affinity purification buffer and Wash Buffer (2% SDS + 6M urea +150 mM NaCl) and eluted in 2% SDS, 6M urea, 30mM biotin, and 2M thiourea for 15 min at room temperature and 15 min at 96 °C. Intermediate samples were collected after pre-clearing lysates and acetone precipitation. For proteomics, beads were washed twice each with affinity purification buffer and wash buffer (2% SDS, 6M urea, 150 mM NaCl) and once with PBS to remove SDS. Beads were frozen at −80 °C until processing.

#### Mass spectrometry

Sample preparation, mass spectrometry analysis, bioinformatics, and data evaluation for quantitative proteomics experiments were performed in collaboration with the Indiana University Proteomics Center for Proteome Analysis at the Indiana University School of Medicine, similar to previously published protocols. ^94^ *Sample Preparation*: On-bead samples were submitted to the IUSM Center for proteome analysis, where proteins were denatured in 8 M urea, 100 mM Tris-HCl, pH 8.5, and reduced with 5 mM tris(2-carboxyethyl) phosphine hydrochloride (TCEP, Sigma-Aldrich Cat No: C4706) for 30 min at room temperature. Samples were then alkylated with 10 mM chloroacetamide (CAA, Sigma Aldrich C0267) for 30 min at room temperature in the dark, prior to dilution with 50 mM Tris-HCl, pH 8.5 to a final urea concentration of 2 M for Trypsin/Lys-C based overnight protein digestion at 37 °C (0.5 μg protease, Mass Spectrometry grade, Promega V5072). *Peptide Purification and Labeling*: Digestions were acidified with trifluoroacetic acid (TFA, 0.5% v/v) and desalted on Pierce C18 spin columns (Thermo Fisher Cat No: 89870) with a wash of 0.5% TFA followed by elution in 70% acetonitrile 0.1% formic acid (FA). *Nano-LC-MS/MS*: Mass spectrometry was performed utilizing an EASY-nLC 1200 HPLC system (SCR: 014993, Thermo Fisher Scientific) coupled to Exploris 480 mass spectrometer with FAIMSpro interface (Thermo Fisher Scientific). 1/5^th^ of each fraction was loaded onto a 25 cm EasySpray column (ES902 Thermo Fisher Scientific) at 350 nL/min. The gradient was held at 5% B for 5 min (Mobile phases A: 0.1% formic acid (FA), water; B: 0.1% FA, 80% Acetonitrile (Thermo Fisher Scientific Cat No: LS122500)), then increased from 4 to 30%B over 98 min; 30–80% B over 10 min; held at 80% for 2 min; and dropping from 80 to 4% B over the final 5 min. The mass spectrometer was operated in positive ion mode, default charge state of 2, advanced peak determination on, and lock mass of 445.12003. Three FAIMS CVs were utilized (−40 CV; −55 CV; −70CV) each with a cycle time of 1.3 s and with identical MS and MS2 parameters. Precursor scans (m/z 375–1500) were done with an orbitrap resolution of 120000, RF lens% 40, automatic maximum inject time, standard AGC target, minimum MS2 intensity threshold of 5e3, MIPS mode to peptide, including charges of 2–7 for fragmentation with 30 s dynamic exclusion. MS2 scans were performed with a quadrupole isolation window of 1.6 m/z, 30% HCD CE, 15000 resolution, standard AGC target, automatic maximum IT, fixed first mass of 110 m/z. *Mass spectrometry Data Analysis*: Resulting RAW files were analyzed in Proteome Discover 2.5 (Thermo Fisher Scientific) with either a *Mus musculus* proteome (downloaded 010917, 49922 entries) or a *Homo sapiens* reference proteome FASTA (downloaded from Uniprot 051322 with 78806 entries) plus common contaminants (73 entries). ^95^ SEQUEST HT searches were conducted with a maximum number of 3 missed cleavages; precursor mass tolerance of 10 ppm, and a fragment mass tolerance of 0.02 Da. Static modifications used for the search were carbamidomethylation on cysteine (C). Dynamic modifications included oxidation of methionine (M), deamidation of asparagine or arginine, phosphorylation on serine, threonine, or tyrosine, and acetylation, methionine loss, or methionine loss plus acetylation on protein N-termini. Percolator False Discovery Rate was set to a strict peptide spectral match FDR setting of 0.01 and a relaxed setting of 0.05. Results were loaded into Scaffold Q + S 5.2.2 (Proteome Software) for viewing. Proteomics data are provided in Table S1.

#### siRNA transfections

For siRNA experiments, MIN6 β-cells were reverse transfected. Briefly, cells were trypsinized, counted, and plated at 8e5 cells per well of a 12-well tissue culture dish. Simultaneously, 25pmol of siRNA (ON-TARGETplus SMART pools: mouse *Vdac1* (L-047345–00-0005), non-targeting pool (D-001810–10-20), Horizon Discovery) was complexed with Lipofectamine RNAiMax (2.5 μL) for 5 min at room temperature in serum-free DMEM in a total volume of 100 μL. Complexed siRNAs were added directly to plated cells and cultured overnight, and the medium was changed the next morning. Cells were cultured for 48 h before harvesting cell lysates for downstream analysis.

#### Cellular thermal shift assays (CETSA)

MIN6 cells were cultured in 10cm dishes and pre-treated with either media containing DMSO or 10μM SW016789 for 30 min. The cells are trypsinized, counted, spun down and resuspended in 500μL PBS containing either DMSO or 10μM SW016789. 50μL cell suspension is aliquoted into a PCR strip tube, incubated for 5 min at RT, and heated in a thermocycler for 3 min. After cooling down, 50μL 2x lysis buffer (0.5% Triton X-100, 20% Glycerol, 274 mM NaCl, 50 mM HEPES pH 7.4, 2mM NaVO4, 2mM EGTA, 2mM EDTA, 20mM NAPP, 100mM NaF) is added to each tube of the PCR strip and transferred to a 1.5mL microcentrifuge tube and placed on ice for 20 min for cell lysis to occur. The tubes are centrifuged at 20,000 × *g* at 4C for 20 min to pellet out the aggregated insoluble material.

#### Transcriptomics and data processing

RNA quality control and downstream sequencing was performed by the Center for Medical Genomics at Indiana University School of Medicine. Total RNA samples were first evaluated for their quantity and quality using Agilent Bioanalyzer 2100. All samples were good quality with RIN (RNA Integrity Number) greater than 9. 100 nanograms of total RNA was used for library preparation with the KAPA mRNA Hyperprep Kit (KK8581) on Biomek following the manufacturer’s protocol. Each uniquely dual-indexed library was quantified and quality accessed by Qubit and Agilent TapeStation, and multiple libraries were pooled in equal molarity. The pooled libraries were sequenced with 2 × 100bp paired-end configuration on an Illumina NovaSeq 6000 sequencer using the v1.5 reagent kit. Samples had an average read depth of ~52.7 million reads/sample. The sequencing reads were first quality-checked using FastQC (v.0.11.5, Babraham Bioinformatics, Cambridge, UK) for quality control. The sequence data were then mapped to either the mouse reference genome mm10, the human reference genome hg38, or the rat reference genome rn6 using the RNA-seq aligner STAR (v.2.710a) ^82^ with the following parameter: “–outSAMmapqUnique 60”. To evaluate the quality of the RNA-seq data, the number of reads that fell into different annotated regions (exonic, intronic, splicing junction, intergenic, promoter, UTR, etc.) of the reference genome was assessed using bam-stats (from NGSUtilsJ v.0.4.17). ^83^ Uniquely mapped reads were used to quantify the gene level expression employing featureCounts (subread v.2.0.3) ^84^ with the following parameters: “-s 2 -p –countReadPairs Q 10”. Transcripts per million (TPM) were calculated using length values determined by using the “makeTxDbFromGFF” and “exonsBy” functions in the “GenomicFeatures” library and the “reduce” function in the “GenomicRanges” library in R to find the length of the union of non-over-lapping exons for each gene. ^96^ Raw fastq, read count table, and TPMs are available on NCBI Gene Expression Omnibus (GEO) under accession number GSE194200. Read count table and TPMs are also provided as a supplemental table (Table S2). To assess Vdac1 expression across multiple endocrine cell lines we mined data from NCBI GEO accession GSE238017.

#### Differential expression analysis and temporal clustering by DPGP and WGCNA

Differential gene expression analysis was performed with edgeR by two approaches. First, SW016789 and thapsigargin-treated samples were compared to their respective DMSO time points to identify differentially expressed genes at each time point. A matrix was exported with all results of these pairwise analyses (Table S2). In parallel, the SW016789 and thapsigargin samples were separately analyzed using an edgeR time course analysis which identified genes that were differentially expressed overall during the course of the experiment. After time course analysis in edgeR, a matrix was exported containing the gene names and log _2_ FC values for all genes with |log_2_ FC| > 1 and adj.p.val <0.05 for downstream analysis (Table S2). *DPGP:* Dirichlet-Process Gaussian-Process mixture model (DPGP) was used to cluster genes according to their temporal expression patterns across the time course using an unbiased algorithm. ^36^ DPGP improves upon other clustering methods like hierarchical clustering, k-means clustering, and self-organizing maps in that it does not require the user to specify the number of clusters and addresses the time series dependency issues. The number of clusters is determined by the algorithm using the input data. The resulting clusters represent different sets of response types that can be further explored to reveal insights into important gene regulatory pathways. DPGP runs most efficiently using a matrix of differentially expressed genes already filtered by log_2_ FC and significance. To run DPGP, a Docker container was created to install DPGP and the required package versions in Python 2.7 (https://github.com/PrincetonUniversity/DP_GP_cluster). DPGP was run in this Docker environment using the following Linux commands: “DP_GP_cluster.py -i input/final_data_matrix_SW_lfc1. txt -o output/output_SW -p svg –plot –fast” and “DP_GP_cluster.py -i input/final_data_matrix_Tg_lfc1.txt -o output/output_Tg -p svg –plot –fast”. Plotted outputs from DPGP are in panels that contain transparent red lines for the expression of each individual gene within the cluster, the cluster mean, and a ribbon twice the standard deviation about the cluster means. *WGCNA*: Weighted-gene co-expression network analysis (WGCNA) was applied to the time course transcriptomics data. ^79,97^ A distinction between WGCNA and DPGP is that WGCNA uses the entire unprocessed read count table from the RNAseq time course. This means additional sets of genes may be discovered that did not achieve statistical significance on their own in edgeR. A set of genes all changing in the same direction, even if each individual gene is not significant, may still indicate important pathways involved in the response to SW016789 or thapsigargin. We used WGCNA parameters similar to previous applications in rodent islets. ^98^ For module identification, the expression of 15,000 of the most abundant transcripts from all samples was included. A “signed” network was constructed, yielding modules where all transcripts are positively correlated, with a minimum module size of 20, and a soft thresholding power of 12. The first principal component, or module eigengene (ME) was computed for each module and used to illustrate the expression pattern across samples. Importantly, WGCNA provides an unsupervised analysis of the correlation structure of the transcripts across all samples, with no gene annotation or sample identification information included. Gene set enrichment analysis of genes within each module was conducted using Enrichr-KG, ^80^ yielding FDR-adjusted enrichment across multiple gene set libraries, including KEGG, GWAS catalog, and Gene Ontology (GO). Of the 15,000 transcripts used for module identification, ~14,500 (~96%) were assigned to a module, indicating a high degree of overall correlation structure among the samples. A total of 20 modules were identified, with the number of transcripts per module ranging from 3613 (turquoise) to 43 (royalblue). Among the 20 modules, 16 were significantly enriched in one or more of the Enrichr-KG libraries. WGCNA analysis results are provided in Table S3. To analyze the hypersecretion gene signature identified via DPGP and WGCNA, enrichment analyses, comparisons to DXO-treated mouse islet RNAseq, ^26^ and plot generation were completed using RStudio (R version 4.4.1) including ggplot2, ^81^ ComplexHeatmap, ^86^ ComplexUpset, ^87^ and VennDiagram.^88^ Venn diagrams were also created using molbiotools.com/listcompare.php (Figures S4C and S4D). Gene sets were analyzed with Enrichr ^99^ or Enrichr-KG ^80^ to determine enriched Biological Process GO terms. Outputs are provided in Table S3 and Table S4. *Construction of directed graph of module relationships:* To identify relationships between WGCNA co-expression modules identified from the transcriptomic sequencing of MIN6 cells, *cor.test()* in R was used to compute all pairwise correlations among the module eigengenes (ME). Only modules that enriched for one or more pathways were considered, yielding 15 modules for network construction. Network nodes represent individual modules with the top-enriched term from *Enrich-R* indicated. A full list of enriched terms for each module is provided in Table S3. ME-ME correlation values were used to construct a directed network in *Cytoscape*, where network edges depict correlation values that ranged from ~|0.4| to ~|0.9|, corresponding to P-values of ~0.01 to 10 ^−15^, respectively. Edge color depicts positive vs. negative correlation (red, positive; blue, negative), and thickness indicates the magnitude of correlation, thicker edges showing stronger relationships.

#### Human pancreas tissue staining and microscopy

FFPE tissue blocks were processed into 5 μm sections and mounted on glass slides at the Indiana University School of Medicine Histology Lab Service Core. Slides were deparaffinized by xylene and ethanol washes. Antigen retrieval was performed by heating for 40 min in an Epitope Retrival Steamer with slides submerged in Epitope Retrieval Solution (IHC-Tek). Subsequently, slides were placed onto disposable immunostaining coverplates and inserted into the Sequenza slide rack (Ted Pella/EMS) for washing, blocking, and antibody incubations. After three 10 min washes in IHC wash buffer (0.1% Triton X-100 and 0.01% sodium azide in PBS pH 7.4), slides were blocked for 1 h at room temperature in normal donkey serum (NDS) block solution (5% donkey serum, 1% bovine serum albumin, 0.3% Triton X-100, 0.05% Tween 20, and 0.05% sodium azide in PBS pH 7.4). Slides were then incubated overnight at 4 °C with primary antibodies (see Key Resources Table) diluted in NDS block solution. Antibodies to SEL1L, HRD1, and DERL3, while not previously validated in human pancreas for IHC, have been validated by others (anti-SEL1L, ^100^ anti-HRD1, ^101^ anti-DERL3 (Abcam ab78233 datasheet and Human Protein Atlas^102^)). After three washes in IHC wash buffer 200 μL each), slides were incubated in secondary antibodies in NDS block solution for 1 h at room temperature. The washed slides were mounted in polyvinyl alcohol (PVA) mounting medium (5% PVA, 10% glycerol, 50mM Tris pH 9.0, 0.01% sodium azide) with DAPI (300 nM) added and imaged on Zeiss LSM710 confocal microscope equipped with a Plan-Apochromat 20x/0.8 objective (#420650–9901). Images were processed in the Zeiss Zen software to add scale bars, set coloration for channels, and generate merged images. Scale bars indicate 50 μm. Intensities of SEL1L, HRD1, and DERL3 staining within individual β-cells from 4 to 5 different imaged regions per donor, and the SEL1L/HRD1 and SEL1L/DERL3 ratio calculations were performed using CellProfiler. ^90,103^ Data were processed further in R to generate smoothed histogram plots using geom_density(). Statistical differences in distributions were determined using the non-parametric Kolmogorov-Smirnov (K-S) test. Normality of distribution was assessed by Q-Q tests.

#### Relative gene expression measurements by qPCR

For drug treatments, human islets (50–75) were hand-picked under a dissection microscope and transferred to low-binding 1.5 mL tubes (50 islets/tube). Islets were cultured 24 h in 500 μL of complete CMRL-1066 medium containing indicated drug treatments. Islets were harvested for RNA isolation using the Quick-RNA Microprep (Zymo). Human islet characteristics and donor information are listed in Table S6. MIN6 RNA was isolated using the Aurum Total RNA Mini kit (Bio-Rad). Briefly, after indicated treatments, medium was removed from cells and lysis buffer (with β-mercaptoethanol) was added to cells. Cells were scraped, lysates transferred to 1.5 mL tubes on ice and then transferred to −80 °C for storage. Samples were processed according to the kit manufacturer’s instructions, including on-column digestion with RNase-free DNase. RNA concentration was measured using an Implen Nanophotometer or Nanodrop spectrophotometer and verified to have A_260/280_ ratios >2.0. 1000 ng of RNA was converted into cDNA using the iScript cDNA synthesis kit (Bio-Rad) following manufacturer instructions and the resulting cDNA was diluted 20-fold with water. One μL of diluted cDNA was used in 10 μL qPCR reactions using 2X SYBR Bio-Rad master mix and 250 nM of each primer. Reactions were run in 384-well format on the QuantStudio 5 (Thermo). qPCR data was analyzed using ACTB and VAPA as reference genes. ^78^ Relative expression was calculated by the 2^−ΔΔCt^ method.

#### Cell stress assays

To measure cells going through apoptosis we measured caspase activity with the Caspase3/7-Glo assay (Promega). The Sartorius Incucyte S3 was used to measure the induction of ER stress over time in EndoC-βH1 cells. For this, we transduced cells with a BacMam system expressing SantakaRed constitutively and mNeonGreen under the control of IRE1-mediated intron splicing according to the manufacturer instructions (Montana Molecular). Green and red objects were identified and quantified in the Incucyte software and changes in the GFP+/RFP+ objects over time were exported and plotted in Prism. For area-under-the-curve (AUC) analysis, a baseline of 0.6 was used, based on the steady state level achieved by the end of the time course in DMSO. We ignored any peaks that were either less than 20% of the distance from minimum to maximum Y or were defined by fewer than 10 adjacent points.

#### Lysate generation, SDS-PAGE, and immunoblotting

To generate lysates for immunoblotting, cells were lysed in 1% NP40 lysis buffer (25 mM HEPES, pH 7.4, 1% Nonidet P-40, 10% glycerol, 137 mM NaCl, 1 mM EDTA, 1 mM EGTA, 50 mM sodium fluoride, 10 mM sodium pyrophosphate, 1 mM sodium orthovanadate, 1 mM phenylmethylsulfonyl fluoride, 10 μg/mL aprotinin, 1 μg/mL pepstatin, 5 μg/mL leupeptin), rotated (10 min, 4 °C), and centrifuged (10,000 × g; 10 min; 4 °C), or lysed in RIPA buffer (50 mM HEPES, pH 7.4, 1% Nonidet P-40, 0.5% sodium deoxycholate, 0.1% sodium dodecylsulfate, 150 mM NaCl, 1 mM EDTA, 1 mM EGTA, 50 mM sodium fluoride, 10 mM sodium pyrophosphate, 1 mM sodium orthovanadate, 1 mM phenylmethylsulfonyl fluoride, 10 μg/mL aprotinin, 1 μg/mL pepstatin, 5 μg/mL leupeptin), sonicated 5 min (30s duty cycle), and stored at −80 °C. 40–50 μg of cleared cell lysates were separated on 4–20% gradient gels (MiniProtean or Criterion TGX, Bio-Rad) by SDS-PAGE and transferred to nitrocellulose for immunoblotting. All membranes were blocked in Odyssey blocking buffer (Licor) diluted in TBS-T (20 mM Tris-HCl pH 7.6, 150 mM NaCl, 0.1% Tween 20) for 1 h before overnight incubation with primary antibodies diluted in blocking buffer. After three 10 min washes in TBS-T, membranes were incubated with fluorescent secondary antibodies 1 h at room temperature. After three 10 min washes in TBS-T, membranes were imaged on a Licor Odyssey scanner.

#### Mouse oral glucose tolerance testing

Twelve healthy wild-type male C57BL6/J mice aged 8–9 weeks were fasted for 4 h (5–9a.m.). The mice were injected intraperitoneally with either SW016789 (5 mg/kg) (*N* = 6) or and equal volume of vehicle (20% PEG300 in saline) (*N* = 6). Treatments were alternated so that half of each litter received vehicle or SW016789. After 10 min a zero timepoint bleed was taken followed by an oral gavage of 10% dextrose at 2 g/kg. Bleeds were taken at 5, 15, 30, 60 and 120 min, centrifuged, and plasma glucose was measured using Fujifilm Autokit Glucose.

### QUANTIFICATION AND STATISTICAL ANALYSIS

For time course RNAseq analysis, edgeR was used in R to assess significance by adjusted *p*-value of <0.05. Quantitated data are expressed as mean ± SD. All other data were evaluated using one-way or two-way ANOVA with the multiple comparisons test indicated in the figure legend, or Kolmogorov-Smirnov tests for distribution plots. Q-Q tests were used to assess normality of distribution plots. For all data other than edgeR-analyzed RNAseq, differences were considered significant if *p* < 0.05. Specific tests applied, numbers of samples and independent experiments are referred to in each figure legend. Statistical testing and graph generation was performed in GraphPad Prism 10 and R 4.4.1, and figure panels were assembled in Adobe Illustrator. Model figures for the graphical abstract (Created in BioRender. Kalwat, M. (2025) https://BioRender.com/83t1f60) and Figure 2G (Created in BioRender. Kalwat, M. (2025) https://BioRender.com/sw6ovh9) were created with BioRender.

## Supplementary Material

1

2

3

4

5

6

SUPPLEMENTAL INFORMATION

Supplemental information can be found online at https://doi.org/10.1016/j.celrep.2025.115834.

## Figures and Tables

**KEY RESOURCES TABLE:** 

REAGENT or RESOURCE	SOURCE	IDENTIFIER

Antibodies		

mouse anti-CHOP (IB: 1:1000)	Cell Signaling Technology	Cat#: 2895. RRID:AB_2089254
mouse anti-PARP (cleaved) (mouse-spec) (IB: 1:1000)	Cell Signaling Technology	Cat#: 9548. RRID:AB_2160592
mouse anti-PARP (cleaved) (human-spec) (IB: 1:1000)	Cell Signaling Technology	Cat#: 32563. RRID:AB_2799024
rabbit anti-cFOS (IB: 1:1000)	Cell Signaling Technology	Cat#: 2250. RRID:AB_2247211
rabbit anti-PHGDH (IB: 1:3000)	ProteinTech	Cat#: 14719-1-AP. RRID:AB_2283938
mouse anti-SEL1L (IB: 1:500; IHC: 1:100)	Santa Cruz	Cat#: sc-377350. RRID:AB_2924977
rabbit anti-HRD1 (SYVN1) (IB: 1:2000; IHC: 1:200)	ProteinTech	Cat#: 13473-1-AP. RRID:AB_2287023
rabbit anti-GRP78/BiP (IB: 1:1000)	Cell Signaling Technology	Cat#: 3177. RRID:AB_2119845
rabbit anti-DNAJC3 (p58IPK, ERdj6) (IB: 1:1000)	Cell Signaling Technology	Cat#: 2940. RRID:AB_2095213
rabbit anti-DNAJB11 (ERdj3) (IB: 1:4000)	ProteinTech	Cat#: 15484-1-AP. RRID:AB_2094400
rabbit anti-VDAC1 (IB: 1:1000)	Cell Signaling Technology	Cat#: 4661. RRID:AB_10557420
mouse anti-Actin (IB: 1:2000)	Sigma	Cat#: A2228. RRID:AB_476697
Mouse anti-Cav1.2 (1:200)	NeuroMab (DHSB)	Cat#: L57/46 RRID:AB_2877240
rabbit anti-Vinculin (IB: 1:1000)	Cell Signaling Technology	Cat#: 4650. RRID:AB_10559207
rabbit anti-eIF2a-pS51 (IB: 1:1000)	Cell Signaling Technology	Cat#: 3398. RRID:AB_2096481
mouse anti-GAPDH (IB: 1:1000)	Cell Signaling Technology	Cat#: 97166. RRID:AB_2756824
rabbit anti-GAPDH (IB: 1:250)	Santa Cruz	Cat#: sc-25778. RRID:AB_10167668
goat anti-Insulin (IHC: 1:300)	Santa Cruz	Cat#: sc-7839. RRID:AB_2296108
mouse anti-Glucagon (IHC: 1:1000)	Sigma	Cat#: G2654. RRID:AB_259852
rabbit anti-DERL3 (IHC: 1:50)	Abcam	Cat#: ab78233. RRID:AB_1566125
DAPI (IHC: 300nM)	Invitrogen	Cat#: D1306.
donkey anti-*anti*-rabbit 680 (IB: 1:10,000)	Licor	Cat#: 92668073. RRID:AB_10954442
donkey anti-*anti*-mouse 800 (IB: 1:10,000)	Licor	Cat#: 92632212. RRID:AB_621847
streptavidin-680 (IB: 1:10,000)	Licor	Cat#: 92568079. RRID:AB_3676093
donkey anti-*anti*-goat 488 (IHC: 1:400)	Jackson Immuno	Cat#: 705545147. RRID:AB_2336933
donkey anti-*anti*-rabbit 594 (IHC: 1:400)	Jackson Immuno	Cat#: 711585152. RRID:AB_2340621
donkey anti-*anti*-mouse 674 (IHC: 1:400)	Jackson Immuno	Cat#: 715605150. RRID:AB_2340862

Bacterial and virus strains		

Green/Red Ratiometric Cell Stress Assay (BacMam)	Montana Molecular	Cat#: U0901G

Biological samples		

Human Pancreas FFPE samples	National Disease Research Interchange	N/A

Chemicals, peptides, and recombinant proteins		

DMEM, high glucose	Corning	Cat#: MT10013CV
β-mercaptoethanol	Sigma	Cat#: M3148
Penicillin/Streptomycin	Sigma	Cat#: P4333
L-Glutamine	Corning	Cat#: MT25005CI
G418 sulfate	GoldBio	Cat#: G-418-10
Trypsin/EDTA	Sigma	Cat#: T3924
Fetal Bovine Serum	Sigma	Cat#: F4135
CMRL-1066	Corning	Cat#: 11530037
DMEM, low gluocse	Corning	Cat#: MT10014CV
BSA, fatty acid-free	GoldBio	Cat#: A-421-500
Sodium selenite	Sigma	Cat#: S1382
Nicotinamide	Sigma	Cat#: 72340
Human transferrin	Sigma	Cat#: T8158
ECM	Sigma	Cat#: E1270
Fibronectin	Sigma	Cat#: F141
Benzonase	Sigma	Cat#: E1014
Biotin-azide-plus	Vector Labs (formerly Click Chemistry Tools)	Cat#: CCT-1488
Sodium ascorbate	Fisher	Cat#: A17759-22
THPTA	Vector Labs (formerly Click Chemistry Tools)	Cat#: CCT-1010
CuSO_4_	Fisher	Cat#: BP346-500
SW016789	Chembridge	Cat#: 5785370
SW016789-A1	Chembridge	Cat#: 5787527
SW016789-A2	Chembridge	Cat#: 6035530
SW016789-A3	Chembridge	Cat#: 6037266
SW016789-A4	Chembridge	Cat#: 5574401
SW016789-A5	Chembridge	Cat#: 6037874
SW016789-A6	Chembridge	Cat#: 5788352
SW016789-A7	Chembridge	Cat#: 6980591
SW016789-A8	Chembridge	Cat#: 6037669
SW016789-A9	Chembridge	Cat#: 5786308
SW016789-A10	Chembridge	Cat#: 5786510
Z6292276622 (Z629)	Enamine	Custom Synthesis
coelenterazine	Nanolight	Cat#: 3035MG
diazoxide	Sigma	Cat#: D9035
fura-2-LR-AM	Fisher	Cat#: 34-491-11SET
nifedipine	Sigma	Cat#: N7634-1G
eeyarestatin I	Cayman	Cat#: 10012609
thapsigargin	Fisher	Cat#: 50-464-293
VBIT4	Selleck	Cat#: S35445
High-binding Streptavidin Agarose beads	Fisher	Cat#: PI20357
Odyssey Blocking Buffer	Li-Cor	Cat#: 927-60003
Lipofectamine RNAiMax	Invitrogen	Cat#: 13778075
Epitope Retrieval Solution	IHC-Tek	Cat#: IW11001L
PEG300	MedChemExpress	Cat#: HY-Y0873

Critical commercial assays		

Caspase 3/7 Glo	Promega	Cat#: G8090
Aurum RNA miniprep kit with DNAse	BioRad	Cat#: 7326820
Quick-RNA Microprep	Zymo	Cat#: R1050
iScript cDNA synthesis kit	BioRad	Cat#: 1708891
2X Sybr qPCR master mix	BioRad	Cat#: 1725125
Autokit Glucose	Fujifilm	Cat#: 997-03001
FTA Sample Collection Kit for Human Cell Authentication Service	ATCC	Cat #: 135-XV
FTA Sample Collection Kit for Mouse Cell Authentication Service	ATCC	Cat #: 137-XV

Deposited data		

Time-course transcriptomics in MIN6 cells exposed to DMSO, SW016789, or thapsigargin	This study	GEO: GSE194200
Endocrine cell ER stress conservation	NCBI GEO	GEO: GSE238017
Analyzed RNAseq data from DXO-treated mouse islets	Pelligra et al.^26^	Mendeley Data: https://doi.org/10.17632/g4bdvw6czr.2
Analyzed RNAseq data from islets of mice treated 10 days with glibenclamide	York et al.^62^	Figshare: https://doi.org/10.2337/figshare.28003664
Analyzed RNAseq data from FACS-purified β-cells of *Abcc8* knockout mice.	Stancill et al.^34^	ArrayExpress: E-MTAB-4726
Proteomics data associated with SW016789 target identification	This study	ProteomeXchange: PXD050590

Experimental models: Cell lines		

MIN6	Miyazaki et al.^75^	RRID: CVCL_0431
EndoC-βH1	Human Cell Design^76^	RRID: CVCL_L909

Experimental models: Organisms/strains		

C57BL6/J mice	Jackson Labs	RRID: IMSR_JAX:000664

Oligonucleotides		

Human *XBP1S*: Fwd: 5′ GCTGAGTCCGCAGCAGGT 3′ Rev: 5′ CTGGGTCCAAGTTGTCCAGAAT 3′	Genewiz	Yoon et al.^77^
Human *SST* Fwd: 5′ ACCCAACCAGACGGAGAATGA 3′ Rev: 5′ GCCGGGTTTGAGTTAGCAGA 3′	Genewiz	Primerbank: 71979669c1
Human *DDIT3* Fwd: 5′ GGAAACAGAGTGGTCATTCCC 3′ Rev: 5′ CTGCTTGAGCCGTTCATTCTC 3′	Genewiz	Primerbank: 304282228c1
Human *HSPA5* Fwd: 5′ ATCAACGAGCCTACGGCAG 3′ Rev: 5′ TCCATGACACGCTGGTCAAA 3′	Genewiz	NCBI Primerblast: NM_005347.5
Human *VAPA* Fwd: 5′ TACCGAAACAAGGAAACTAATGGAA 3′ Rev: 5′ GCCTTAAACCTTCATCTCTCAGGT 3′	Genewiz	Alvelos et al.^78^
Human *ACTB* Fwd: 5′ CTGTACGCCAACACAGTGCT 3′ Rev: 5′ GCTCAGGAGGAGCAATGATC 3′	Genewiz	Alvelos et al.^78^
Mouse *Vdac1* ON-TARGETplus SMART pool	Horizon Discovery	Cat#: L-047345-00-0005
non-targeting ON-TARGETplus SMART pool	Horizon Discovery	Cat#: D-001810-10-20

Software and algorithms		

Gen5	Agilent BioTek	N/A
Prism 10	GraphPad	N/A
QuantStudio v1.3	Thermo	N/A
Scaffold Q + S 5.2.2	Proteome Software	N/A
Proteome Discover^™^ 2.5	Thermo Fisher Scientific	N/A
Incucyte S3 software	Sartorius	N/A
Python 2.7		N/A
Dirichlet Process-Gaussian Process (DPGP)	McDowell et al.^36^	Github: https://github.com/PrincetonUniversity/DP_GP_cluster
Weighted gene correlation network analysis	Langfelder et al.^79^	N/A
Enrichr-KG	Evangelista et al.^80^	N/A
RStudio (R version 4.4.1)	Posit	N/A
ggplot2	Wickham^81^	N/A
FastQC v.0.11.5	Babraham Bioinformatics	N/A
STAR v.2.710a	Dobin et al.^82^	N/A
bam-stats v.0.4.17 (NGSUtilsJ)	Breese et al.^83^	N/A
featureCounts (subread v.2.0.3)	Liao et al.^84^	N/A
edgeR v4.4.0	Chen et al.^85^	N/A
ComplexHeatmap	Gu^86^	N/A
ComplexUpset	Krassowski, M.^87^	N/A
VennDiagram	Chen et al.^88^	N/A
Cytoscape 3	Shannon et al.^89^	N/A
Zen 2.6 lite	Zeiss	N/A
CellProfiler 4.2.7	Stirling et al.^90^	N/A
BioRender	Biorender.com	N/A
Illustrator	Adobe	N/A
R scripts for processing RNAseq data and generating heatmaps	This study	Github: https://github.com/kalwatlab/hypersecretion-timecourse

Other		

96 well white assay plates	Thomas Scientific	Cat#: 1185U49
Mini-protean 4–20% SDS-PAGE precast gels	BioRad	Cat#: 4561095
Criterion TGX 4–20% SDS-PAGE precast gels	BioRad	Cat#: 5671093

## Highlights

- VDAC1 is a pharmacological target for insulin hypersecretion
- Hypersecretion induces a unique transcriptomic signature in β cells
- ER-associated degradation (ERAD) may protect β cells during insulin hypersecretion
