# Supplementary material for: VDAC1 is a target for pharmacologically induced insulin hypersecretion in β cells
Source: Cell Rep. Author manuscript; Available in PMC 2025 Jul 16. (PMC12266945; doi:10.1016/j.celrep.2025.115834)
Supplement: 1 [file NIHMS2092694-supplement-1.pdf]

Cell Reports, Volume *44*

Supplemental information

VDAC1 is a target for pharmacologically induced insulin hypersecretion in β cells

Gitanjali Roy, Andrea Ordóñez, Derk D. Binns, Karina Rodrigues-dos-Santos, Michael B. Kwakye, George C. King, Rachel L. Kuntz, Noyonika Mukherjee, Andrew T. Templin, Zhiyong Tan, Timothy I. Richardson, Emma H. Doud, Amber L. Mosley, Kathryn L. Schueler, Christopher H. Emfinger, Alan D. Attie, Mark P. Keller, Travis S. Johnson, and Michael A. Kalwat

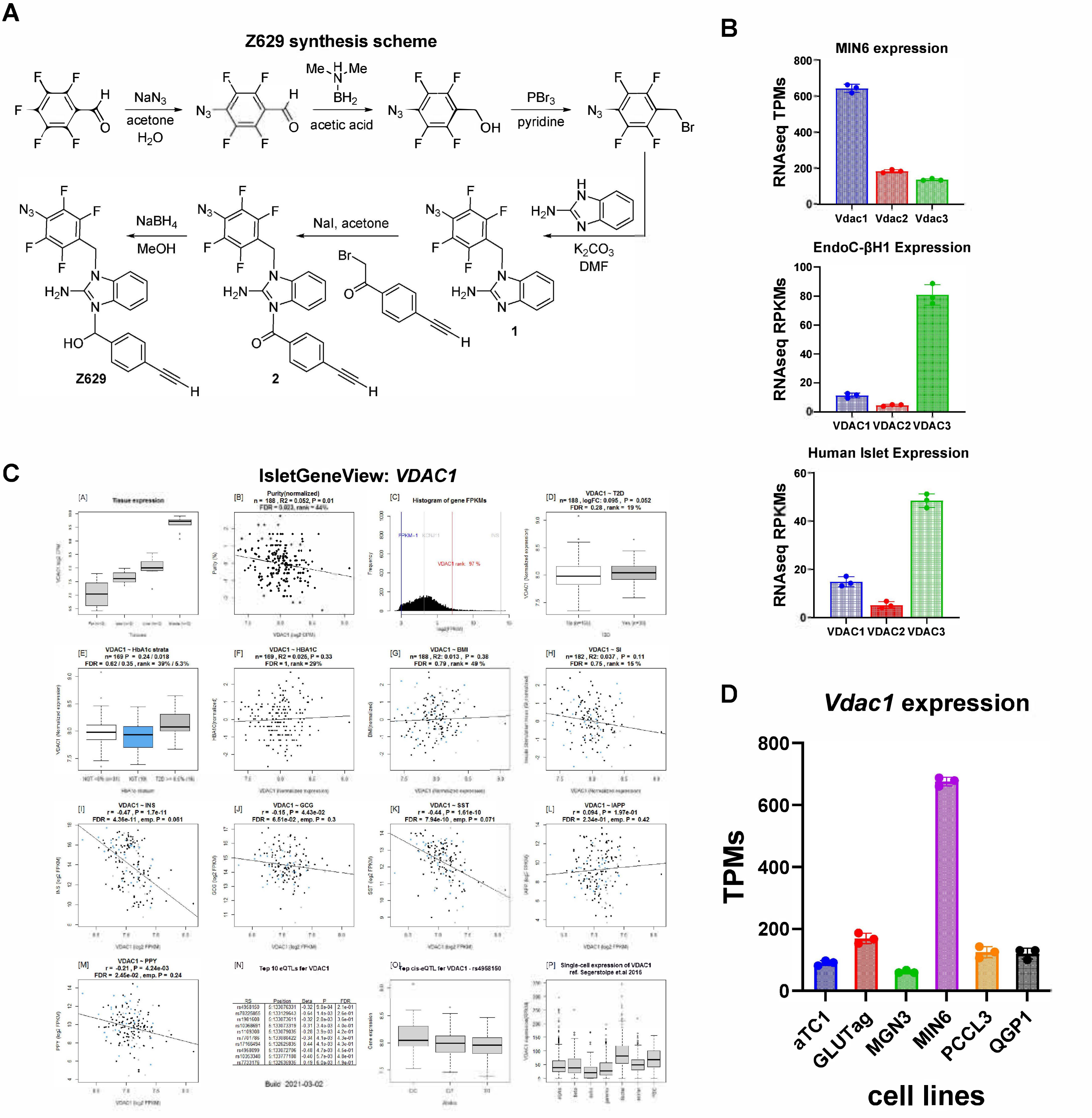

A
Z629 synthesis scheme
NaN3
acetone
H2O
Me
H
N
Me
BH2
acetic acid
PBr3
pyridine
H2N
K2CO3
DMF
NaI, acetone
NaBH4
MeOH
1
2
Z629
B
MIN6 expression
RNAseq TPMs
Vdac1
Vdac2
Vdac3
EndoC-βH1 Expression
RNAseq RPKMs
VDAC1
VDAC2
VDAC3
Human Islet Expression
C
IsletGeneView: VDAC1
Tissue expression
Purity(normalized)
Histogram of gene FPKMs
VDAC1 ~ T2D
VDAC1 ~ HbA1c strata
VDAC1 ~ HBA1C
VDAC1 ~ BMI
VDAC1 ~ SI
VDAC1 ~ INS
VDAC1 ~ GCG
VDAC1 ~ SST
VDAC1 ~ IAPP
VDAC1 ~ PPY
Top 10 eQTLs for VDAC1
Top cis-eQTL for VDAC1 - rs4958150
Single-cell expression of VDAC1
ref. Segerstolpe et.al 2016
Build: 2021-03-02
D
Vdac1 expression
TPMs
aTC1
GLUTag
MGN3
MIN6
PCCL3
QGP1
cell lines

Figure S1

Figure S1. SW016789 photoaffinity probe synthesis and β-cell VDAC expression, Related to Figure 1. A) Synthetic scheme for Z6292276622 (Z629) which incorporates an aryl azide for UV crosslinking and an alkyne for click chemistry. **B)** Expression of *VDAC1*, *VDAC2*, and *VDAC3* in MIN6, EndoC-βH1, and human islets. MIN6 TPMs are from our own RNAseq data (GSE194200). EndoC-βH1 and human islet RPKM data is from Fred RG, et al.[S1]. **C)** IsletGeneView report on VDAC1 expression in a large human islet RNAseq dataset containing non-diabetic and type 2 diabetic donor islets. VDAC1 is well-expressed in islets and is negatively correlated with *INS* expression. **D)** *Vdac1* expression (TPMs) in αTC1-6 (mouse α-cells), GLUTag (mouse L-cells), MGN3 (mouse ghrelin cells), MIN6 (mouse β-cells), PCCL3 (rat thyrocyte cells), and QGP1 (human δ-cell somatostatinoma line).

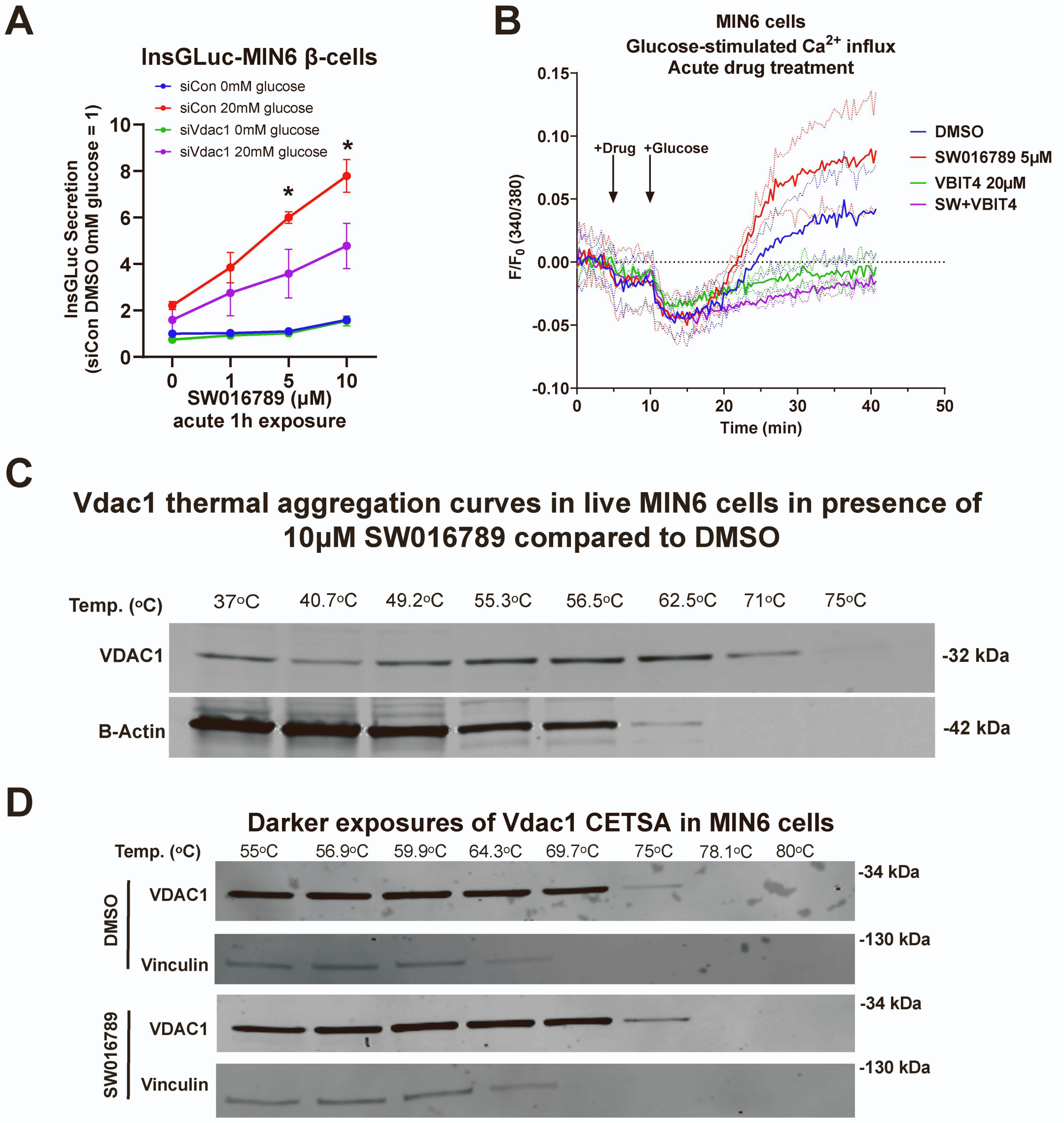
A
InsGLuc-MIN6 β-cells
siCon 0mM glucose
siCon 20mM glucose
siVdac1 0mM glucose
siVdac1 20mM glucose
InsGLuc Secretion (siCon DMSO 0mM glucose = 1)
SW016789 (μM) acute 1h exposure
B
MIN6 cells
Glucose-stimulated Ca2+ influx
Acute drug treatment
+Drug
+Glucose
DMSO
SW016789 5μM
VBIT4 20μM
SW+VBIT4
F/F0 (340/380)
Time (min)
C
Vdac1 thermal aggregation curves in live MIN6 cells in presence of 10μM SW016789 compared to DMSO
Temp. (oC)
37oC
40.7oC
49.2oC
55.3oC
56.5oC
62.5oC
71oC
75oC
VDAC1
B-Actin
-32 kDa
-42 kDa
D
Darker exposures of Vdac1 CETSA in MIN6 cells
Temp. (oC)
55oC
56.9oC
59.9oC
64.3oC
69.7oC
75oC
78.1oC
80oC
DMSO
SW016789
VDAC1
Vinculin
-34 kDa
-130 kDa

Figure S2

Figure S2. VDAC1 is required for full activity of SW016789 and is thermally stabilized by SW016789, Related to Figure 2. A) VDAC1 is required in InsGLuc-MIN6 cells for full secretory response to SW016789 in the presence of 20 mM glucose. Data are the mean ± SD of N=3. *, $P<0.05$ siCon G20 vs siVdac1 G20 by two-way ANOVA with Tukey's multiple comparisons test. **B)** Glucose-stimulated Ca^{2+} influx in MIN6 β-cells treated acutely with SW016789 (SW, 5 μM), VBIT4 (20 μM), or both. Data represent the mean ± SD of two independent experiments. **C)** Thermal stability of endogenous VDAC1 (37°C – 75°C) in the absence of ligand shows stability up to 71˚C compared to β-actin in MIN6 β-cells. **D)** Darker exposures are shown of the same Vdac1 blots from Fig 2E. Vinculin loading control is shown but is not used for Vdac1 normalization. Data are representative of three independent experiments.

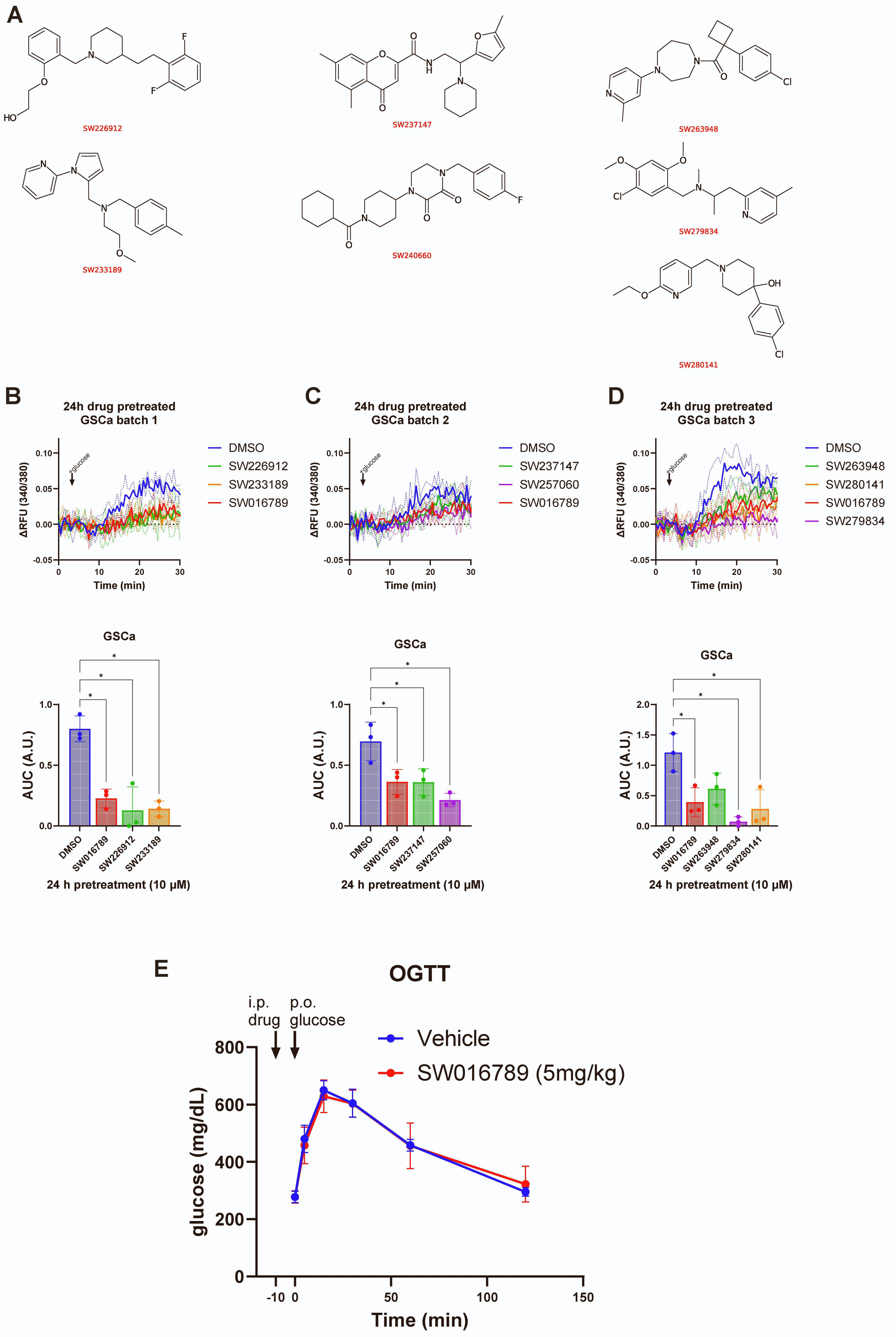
A
SW226912
SW237147
SW263948
SW233189
SW240660
SW279834
SW280141
B
24h drug pretreated
GSCa batch 1
C
24h drug pretreated
GSCa batch 2
D
24h drug pretreated
GSCa batch 3
+glucose
ΔRFU (340/380)
Time (min)
DMSO
SW226912
SW233189
SW016789
SW237147
SW257060
SW263948
SW280141
SW279834
GSCa
AUC (A.U.)
24 h pretreatment (10 μM)
E
OGTT
i.p.
drug
p.o.
glucose
Vehicle
SW016789 (5mg/kg)
glucose (mg/dL)
Time (min)

Figure S3

Figure S3. Diverse small molecule hypersecretion inducers phenocopy SW016789, Related to Figure 3. A) Structures are shown for high-throughput screening hits previously identified [S2]. **B-D)** Glucose-stimulated Ca^{2+} influx after 24 h pretreatment with 5 µM SW016789 as a positive control, or with 10 µM of test compounds in the following panels: **B)** SW226912, SW233189; **C)** SW237147, SW257060; **D)** SW263948, SW280141, or SW279834 . Data are the mean ± SD of N=3 experiments. *, P<0.05 vs DMSO by one-way ANOVA with Dunnett's multiple comparisons test. **E)** Mice were fasted for 4 h, injected i.p. with SW016789 (5 mg/kg) or vehicle (20% PEG300 in saline). 10 min later, baseline blood sample was taken and glucose was gavaged at 2 mg/kg. Blood glucose was measured at baseline (0), 5, 15, 30, 60 and 120 minutes. Data are the mean ± SD of N=6 mice per group.

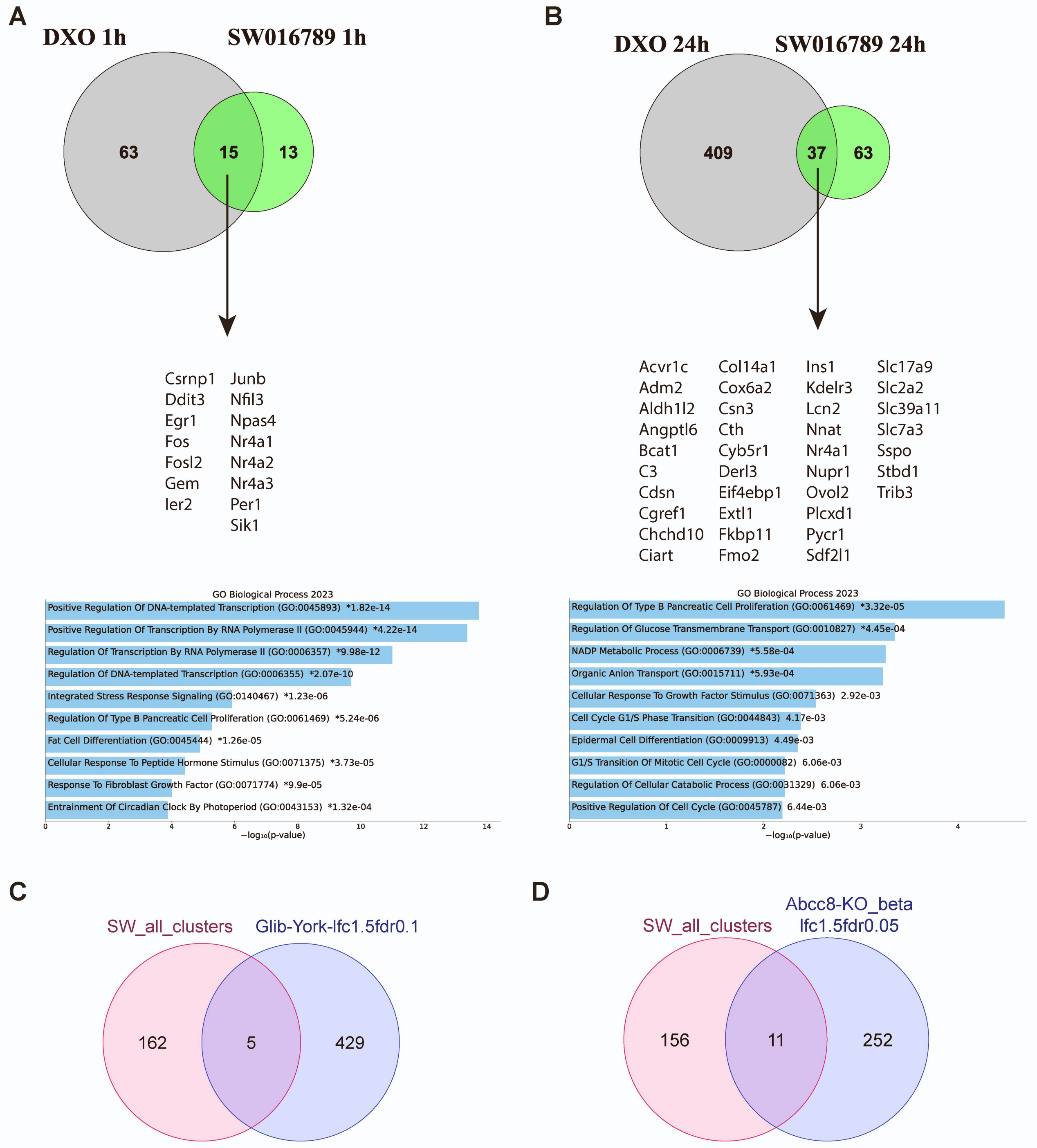

A
DXO 1h
SW016789 1h
63
15
13
Csrnp1
Ddit3
Egr1
Fos
Fosl2
Gem
Ier2
Junb
Nfil3
Npas4
Nr4a1
Nr4a2
Nr4a3
Per1
Sik1
GO Biological Process 2023
Positive Regulation Of DNA-templated Transcription (GO:0045893) *1.82e-14
Positive Regulation Of Transcription By RNA Polymerase II (GO:0045944) *4.22e-14
Regulation Of Transcription By RNA Polymerase II (GO:0006357) *9.98e-12
Regulation Of DNA-templated Transcription (GO:0006355) *2.07e-10
Integrated Stress Response Signaling (GO:0140467) *1.23e-06
Regulation Of Type B Pancreatic Cell Proliferation (GO:0061469) *5.24e-06
Fat Cell Differentiation (GO:0045444) *1.26e-05
Cellular Response To Peptide Hormone Stimulus (GO:0071375) *3.73e-05
Response To Fibroblast Growth Factor (GO:0071774) *9.9e-05
Entrainment Of Circadian Clock By Photoperiod (GO:0043153) *1.32e-04
0 2 4 6 8 10 12 14
−log10(p-value)
B
DXO 24h
SW016789 24h
409
37
63
Acvr1c
Adm2
Aldh1l2
Angptl6
Bcat1
C3
Cdsn
Cgref1
Chchd10
Ciart
Col14a1
Cox6a2
Csn3
Cth
Cyb5r1
Derl3
Eif4ebp1
Extl1
Fkbp11
Fmo2
Ins1
Kdelr3
Lcn2
Nnat
Nr4a1
Nupr1
Ovol2
Plcxd1
Pycr1
Sdf2l1
Slc17a9
Slc2a2
Slc39a11
Slc7a3
Sspo
Stbd1
Trib3
GO Biological Process 2023
Regulation Of Type B Pancreatic Cell Proliferation (GO:0061469) *3.32e-05
Regulation Of Glucose Transmembrane Transport (GO:0010827) *4.45e-04
NADP Metabolic Process (GO:0006739) *5.58e-04
Organic Anion Transport (GO:0015711) *5.93e-04
Cellular Response To Growth Factor Stimulus (GO:0071363) 2.92e-03
Cell Cycle G1/S Phase Transition (GO:0044843) 4.17e-03
Epidermal Cell Differentiation (GO:0009913) 4.49e-03
G1/S Transition Of Mitotic Cell Cycle (GO:0000082) 6.06e-03
Regulation Of Cellular Catabolic Process (GO:0031329) 6.06e-03
Positive Regulation Of Cell Cycle (GO:0045787) 6.44e-03
0 1 2 3 4
−log10(p-value)
C
SW_all_clusters
Glib-York-lfc1.5fdr0.1
162
5
429
D
SW_all_clusters
Abcc8-KO_beta
lfc1.5fdr0.05
156
11
252

Figure S4

Figure S4. Differential gene expression comparison of SW016789 and DXO, Related to Figure 4. A,B) Published transcriptomics data of DXO-treated mouse islets [S3] was compared to our SW016789-treated mouse MIN6 β-cell transcriptomics data at the corresponding 1 h (**A**) and 24 h (**B**) time points. Below the Venn diagrams the overlapping genes are shown (also in **Table S4**) as well as the GO Biological Process enrichment. **C,D)** Differentially-expressed genes from the SW016789 time course transcriptomics were compared to published RNAseq datasets from FACS-purified β-cells of *Abcc8* knockout mice [S4] and from islets of mice treated for 10 days with glibenclamide [S5]. Results of the overlaps are provided in **Table S5**.

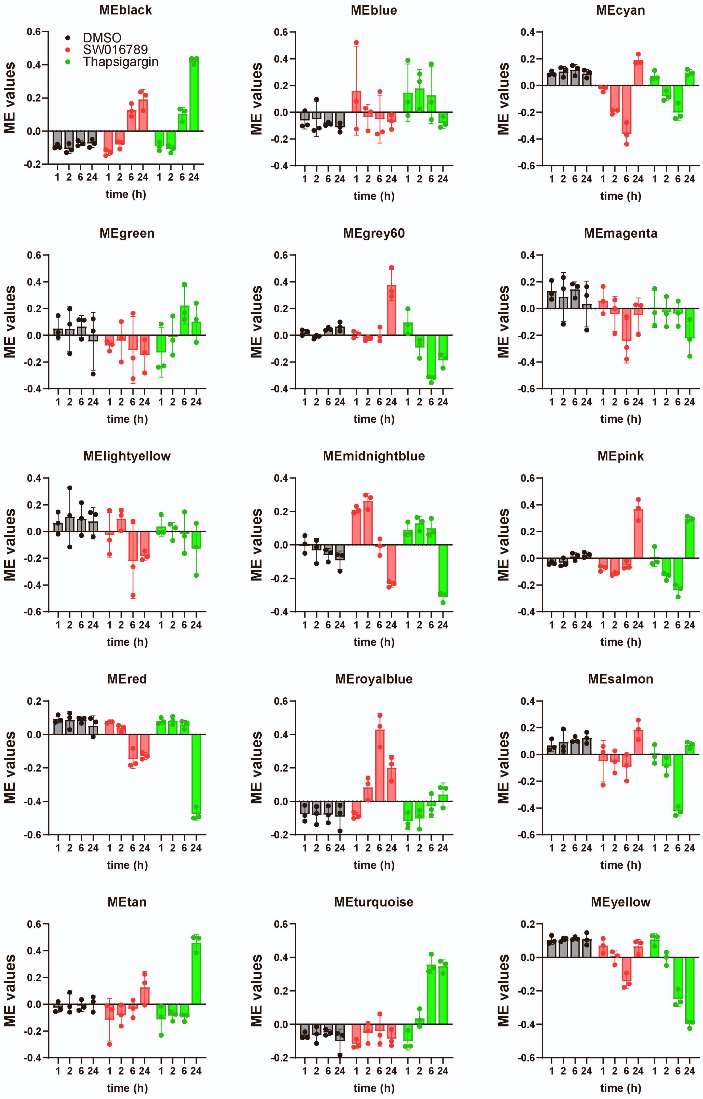
MEblack
DMSO
SW016789
Thapsigargin
MEblue
MEcyan
MEgreen
MEgrey60
MEmagenta
MElightyellow
MEmidnightblue
MEpink
MEred
MEroyalblue
MEsalmon
MEtan
MEturquoise
MEyellow
ME values
time (h)

Figure S5

Figure S5. WGCNA modules identified from hypersecretion and ER stress time course transcriptomics, Related to Figure 5. Bar graphs show the mean ± SD of module eigengene (ME) values for each treatment within each module across the time course.

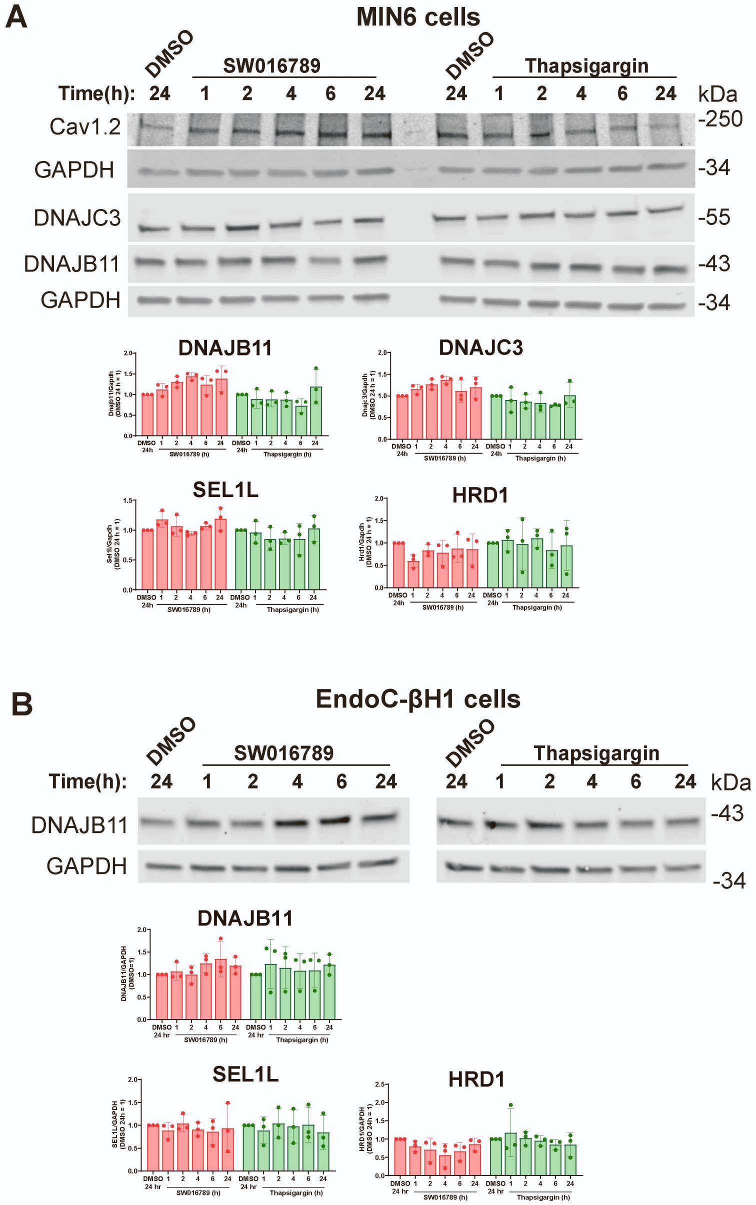

A
MIN6 cells
DMSO
SW016789
DMSO
Thapsigargin
Time(h):
24
1
2
4
6
24
kDa
Cav1.2
GAPDH
DNAJC3
DNAJB11
-250
-34
-55
-43
DNAJB11
DNAJC3
SEL1L
HRD1
Dnajb11/Gapdh (DMSO 24 h = 1)
Dnajc3/Gapdh (DMSO 24 h = 1)
Sel1l/Gapdh (DMSO 24 h = 1)
Hrd1/Gapdh (DMSO 24 h = 1)
DMSO 24h
SW016789 (h)
Thapsigargin (h)
B
EndoC-βH1 cells
DNAJB11/GAPDH (DMSO=1)
SEL1L/GAPDH (DMSO 24h = 1)
HRD1/GAPDH (DMSO 24h = 1)
DMSO 24 hr

Figure S6

Figure S6. Immunoblots for putative proteins responding to hypersecretory stress, Related to Figure 6. A) MIN6 β-cells were treated with SW016789 (5 μM), thapsigargin (100 nM), or DMSO (0.1%) for the indicated times and samples were analyzed by immunoblotting. Quantification is shown for DNAJB11 and DNAJC3. Quantification of SEL1L and HRD1 from **Fig 6D** are also shown. Data are the mean ± SD of N=3. **B)** Human EndoC-βH1 cells were treated the same as in (**A**), except thapsigargin was used at 1 μM. Quantification is shown for DNAJB11. The GAPDH blot shown is the same as the lower blot in Fig 6E only for comparison of loading. Quantification of SEL1L and HRD1 from **Fig 6E** are also shown. Data are the mean ± SD of N=3.

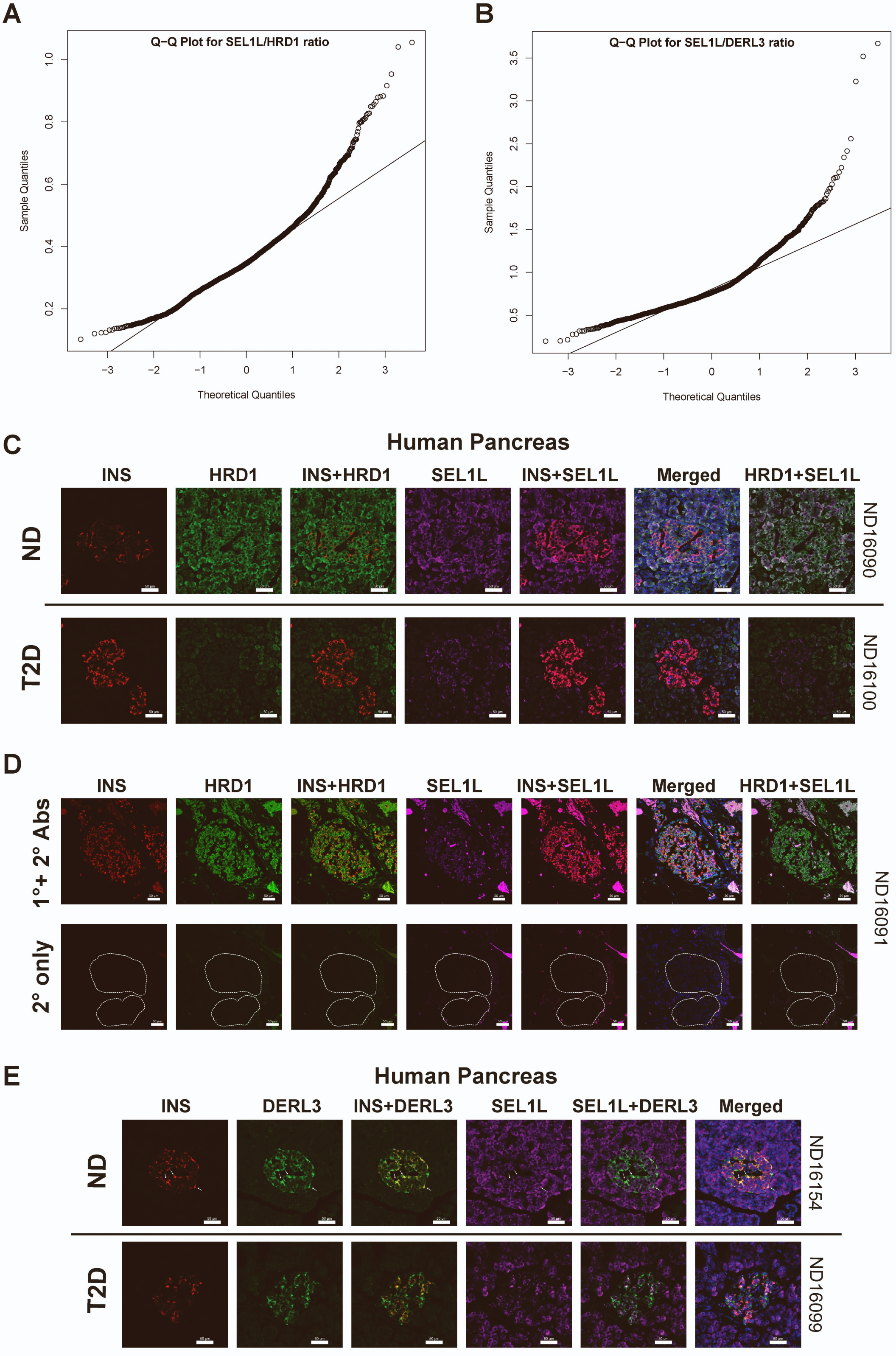
A
Q–Q Plot for SEL1L/HRD1 ratio
Sample Quantiles
Theoretical Quantiles
B
Q–Q Plot for SEL1L/DERL3 ratio
Sample Quantiles
Theoretical Quantiles
C
Human Pancreas
INS
HRD1
INS+HRD1
SEL1L
INS+SEL1L
Merged
HRD1+SEL1L
ND
ND16090
T2D
ND16100
D
INS
HRD1
INS+HRD1
SEL1L
INS+SEL1L
Merged
HRD1+SEL1L
1°+ 2° Abs
2° only
ND16091
E
Human Pancreas
INS
DERL3
INS+DERL3
SEL1L
SEL1L+DERL3
Merged
ND
ND16154
T2D
ND16099

Figure S7

Figure S7. Additional donor and control immunohistochemistry, Related to Figure 7. A) Q-Q tests to assess normality of (**A**) SEL1L/HRD1 and (**B**) SEL1L/DERL3 distribution ratios. The straight diagonal line indicates normality. Both Q-Q plots show a positive skew. **B)** Staining of HRD1 and SEL1L in a third human donor, related to Fig 7A. **C)** Additional staining of HRD1 and SEL1L in a fourth human donor, along with secondary-only antibody controls to support specific staining of pancreatic HRD1 and SEL1L. Outlined islet region was identified by distinct morphology. Images are representative of three different imaged regions. **D)** Staining of INS, DERL3, and SEL1L in a third human donor, related to Fig 7D. All scale bars are 50 μm.

Checklist for reporting human islet preparations used in research

Adapted from Hart NJ, Powers AC (2018) Progress, challenges, and suggestions for using human islets to understand islet biology and human diabetes. Diabetologia https://doi.org/10.1007/s00125-018-4772-2

Islet preparation	1	2	3	4	5	6	7	8[a]
**MANDATORY INFORMATION**								
Unique identifier	SAMN30986138	HP-22278-01	HP-23153-01	HP-23166-01	SAMN34033792			
Donor age (years)	67	69	51	58	39			
Donor sex (M/F)	male	male	Female	Female	Male			
Donor BMI (kg/m^2)	34.4	29.1	26.4	28.8	33.4			
Donor HbA_{1c} or other measure of blood glucose control	5.3%	5.4%	5.3%	5.6%	5.0%			
Origin/source of islets[b]								
Islet isolation centre	Scharp-Lacy (Prodo)	Prodo Labs	Prodo Labs	Prodo Labs	Southern California Islet Cell Resource Center			
Donor history of diabetes? Please select yes/no from drop down list	No	No	No	No	No			
**If Yes, complete the next two lines if this information is available**								
Diabetes duration (years)								
Glucose-lowering therapy at time of death[c]								
**RECOMMENDED INFORMATION**								
Donor cause of death	head trauma	anoxia	stroke	stroke	head trauma			

Warm ischaemia time (h)								
Cold ischaemia time (h)	10h 5m				12h 41m			
Estimated purity (%)	90	95	85	80-85	80			
Estimated viability (%)	95	95	95	95	97			
Total culture time (h)[d]								
Glucose-stimulated insulin secretion (static culture by IIDP)[e]	4.6				2.2			
Handpicked to purity? Please select yes/no from drop down list	Yes	Yes	Yes	Yes	Yes			
Additional notes: Related to Figure:	Fig 1B	Fig 1B	Fig 1B	Fig 1B	Fig 1B			
Additional notes -Culture time prior to shipment	3d 20h				2d 7h			

[a]If you have used more than eight islet preparations, please complete additional forms as necessary
[b]For example, IIDP, ECIT, Alberta IsletCore
[c]Please specify the therapy/therapies
[d]Time of islet culture at the isolation centre, during shipment and at the receiving laboratory
[e]Please specify the test and the results

Table S6. Human islet checklist. Standard checklist containing donor metadata associated with human islet preparations used in this study.

SUPPLEMENTARY REFERENCES

S1. Fred, R.G., Kappe, C., Ameur, A., Cen, J., Bergsten, P., Ravassard, P., Scharfmann, R., and Welsh, N. (2015). Role of the AMP kinase in cytokine-induced human EndoC-betaH1 cell death. Mol Cell Endocrinol *414*, 53-63. 10.1016/j.mce.2015.07.015.

S2. Kalwat, M.A. (2021). High-Throughput Screening for Insulin Secretion Modulators. Methods Mol Biol *2233*, 131-138. 10.1007/978-1-0716-1044-2_9.

S3. Pelligra, A., Mrugala, J., Griess, K., Kirschner, P., Nortmann, O., Bartosinska, B., Koster, A., Krupenko, N.I., Gebel, D., Westhoff, P., et al. (2023). Pancreatic islet protection at the expense of secretory function involves serine-linked mitochondrial one-carbon metabolism. Cell Rep *42*, 112615. 10.1016/j.celrep.2023.112615.

S4. Stancill, J.S., Cartailler, J.P., Clayton, H.W., O'Connor, J.T., Dickerson, M.T., Dadi, P.K., Osipovich, A.B., Jacobson, D.A., and Magnuson, M.A. (2017). Chronic beta-Cell Depolarization Impairs beta-Cell Identity by Disrupting a Network of Ca(2+)-Regulated Genes. Diabetes *66*, 2175-2187. 10.2337/db16-1355.

S5. York, N.W., Yan, Z., Osipovich, A.B., Tate, A., Patel, S., Piston, D.W., Magnuson, M.A., Remedi, M.S., and Nichols, C.G. (2025). Loss of beta-Cell KATP Reduces Ca2+ Sensitivity of Insulin Secretion and Trpm5 Expression. Diabetes *74*, 376-383. 10.2337/db24-0650.
